# Aptamer-enabled uptake of small molecule ligands

**DOI:** 10.1038/s41598-018-33887-w

**Published:** 2018-10-24

**Authors:** Supipi Liyamali Auwardt, Yeon-Jung Seo, Muslum Ilgu, Judhajeet Ray, Robert R. Feldges, Shambhavi Shubham, Lee Bendickson, Howard A. Levine, Marit Nilsen-Hamilton

**Affiliations:** 1 Iowa State University, Ames, IA USA; 2 Ames National Laboratory, Ames, IA USA; 3 Aptalogic Inc., Ames, IA USA; 4 Present Address: Middle East Technical University, Ankara, Turkey; 5 Present Address: Cornell University, Ithaca, NY USA; 6 Present Address: Integrated DNA Technologies, Coralville, IA USA

## Abstract

The relative ease of isolating aptamers with high specificity for target molecules suggests that molecular recognition may be common in the folds of natural RNAs. We show here that, when expressed in cells, aptamers can increase the intracellular concentrations of their small molecule ligands. We have named these aptamers as DRAGINs (Drug Binding Aptamers for Growing Intracellular Numbers). The DRAGIN property, assessed here by the ability to enhance the toxicity of their ligands, was found for some, but not all, aminoglycoside aptamers. One aptamer protected cells against killing by its ligand. Another aptamer promoted killing as a singlemer and protected against killing as a tandemer. Based on a mathematical model, cell protection vs. killing is proposed as governed by aptamer affinity and access to the inner surface of the cell membrane, with the latter being a critical determinant. With RNA molecules proposed as the earliest functional polymers to drive the evolution of life, we suggest that RNA aptamer-like structures present in primitive cells might have selectively concentrated precursors for polymer synthesis. Riboswitches may be the evolved forms of these ancient aptamer-like “nutrient procurers”. Aptamers with DRAGIN capability in the modern world could be applied for imaging cells, in synthetic cell constructs, or to draw drugs into cells to make “undruggable” targets accessible to small molecule inhibitors.

## Introduction

Controlling the entrance and exit of small molecules through the cell membrane is critical to cell survival. In modern cells, transmembrane protein transporters promote the entry of essential nutrients for cell proliferation. However, protein transporters were not available to early forms of life that, once surrounded by a hydrophobic membrane, depended on the availability of precursors for their expansion by macromolecular synthesis. Experimental results and logical deductions support the hypothesis that the earliest forms of life relied on RNA molecules to perform replication and other catalysis^1–3^. Initially, life may have evolved within confined inorganic cavities that were possibly located in mineral precipitates such as of iron monosulphide^2^ or on clay, which promotes encapsulation by lipids^4^. It is not clear how long the period of RNA dominance lasted before proteins took over most catalytic functions. But, primitive cells are believed to have evolved through several eras, which included the sequential introduction of functional RNAs, RNPs and proteins culminating in the last universal common ancestor (LUCA), which existed prior to the divergence of the domains of *Bacteria*, *Archaea*, and finally the appearance of *Eukarya*^2,5^. These primitive forms are presumed to have faced the same challenge, as do modern cells, which is to selectively concentrate the precursor molecules required for cell replication and function. The proposed membrane composition of the very early evolving cells, believed to consist of a mixture of fatty acids, glycerol esters and amphiphiles, is more permeable than a membrane of homogeneous lipid composition^6–8^. Small hydrophilic molecules might also have been flipped into the primitive cell by association with charged head-groups of the early membrane constituents^9^. However, neither diffusion nor flipping provides much selectivity to the distribution of small molecules that enter the cell and would have limited the rate of RNA replication in these primitive cells. Once cell membranes evolved to include hydrocarbon chains and ether/ester bonds on a glycerophosphate backbone, the permeability to hydrophilic molecules would have decreased and the cells then needed to evolve transporters to efficiently move needed metabolic precursors into the cell^10^.

Aptamers are small nucleic acids with high affinities and specificities for a particular target molecule or a group of related molecules. They can be obtained *in vitro* by repeated selection and amplification of nucleic acid populations that bind a chosen molecular target^11–14^. Aptamer-like activities are also found as the recognition elements in riboswitches, which are portions of RNAs that regulate transcription or translation differently when unbound compared with when bound to their target ligand. These RNA elements may date back to a period prior to the divergence of the *Bacteria* and *Archaea* domains. This is suggested by the sequence of the TPP riboswitch which is closely related in eubacteria and archaebacteria^15^. Here we consider that aptamers may have been “transporters” of RNA precursors during the earlier era of RNA predominance and later once a primitive membrane had evolved such as in LUCA. As the cellular metabolism and architecture became more complex, aptamers would have also evolved into components of modern-day riboswitches by which they regulate transcription and translation.

We show, experimentally and by mathematical modeling, that RNA aptamers present inside a cell are capable of increasing both the total amounts and the free intracellular concentrations of the small molecules to which they bind. A previous mathematical model based on partial differential equations (PDE), by which we predicted this effect, required that the intracellular aptamer be mobile^16^. Here we describe a related compartmental model in which mobile aptamers have access to their small molecule ligands located near the surface of the inner cell membrane (Math Model, Supplementary Information). By capturing and moving their ligands from the cell membrane where they could readily exit the cell, the aptamers drive the intracellular free ligand concentrations up.

With the development of the cell membrane, a primitive cell would have been advantaged by the ability of aptamers to capture and accumulate substrates for continued replication. The capability of selectively retaining the appropriate small molecule precursors and making them available to the replicating RNA or DNA would have given an evolutionary advantage over other cells. Curiously, many of the identified riboswitches recognize molecules chemically related to nucleic acid precursors.

As well as their proposed existence during early evolution, the ability of aptamers to behave as DRAGINs might be applied in current biology in such contexts as building the minimal cell *in vitro* or to clinical applications such as to enhance the effectiveness of drugs with intracellular targets. In light of their ability to increase the intracellular free concentrations of small molecules, such as drugs, we have dubbed these intracellularly expressed aptamers as DRAGINs (Drug Binding Aptamers for Growing Intracellular Numbers).

## Materials and Methods

### Reagents

Aminoglycosides, neomycin-B, kanamycin-A and Geneticin (G418), were purchased as their sulfate salts from Sigma-Aldrich (St. Louis, MO). Bacterial and yeast expression vectors and their expected expressed RNA products are listed in Tables S1–S3.

### Cell Growth Analysis

BL21 (DE3) star *E*. *coli* (Invitrogen, Eugene, OR) were transformed with the identified bacterial expression vectors and grown for 18 h at 37 °C in Luria-Bertani broth (LB) with 100 μg/mL ampicillin. The cultures were then diluted 100 times and grown at 37 °C to OD = 1.0. The cells were again diluted 100 times (or as identified for the experiment) in LB and grown for 1 h with 1 mM IPTG at 37 °C. Aminoglycosides were added according to the experimental protocol and the cells were grown for 12 h at 37 °C in a Biotek Synergy plate reader sealed with Breathe-Easy sealing membrane (Research Products International Corp). To obtain growth curves, the OD600 was taken every 5, 20 or 60 min, depending on the experiment.

*Saccharomyces cerevisiae* BY4735 were transformed with the identified yeast expression vectors as previously described^17^. The transformed cells were grown in SD-Uracil media in presence of 2% of glucose or raffinose for 19 h at 30 °C. Cells were diluted to A^600^ = 0.2 and Geneticin (G418) was added and grown in SD-Uracil media with 2% glucose or 2% galactose, 1% raffinose in Biotek Synergy plate reader for 30 h at 30 °C. Absorption at 600 nm (A^600^) was determined every 1 h for up to 30 h.

### Northern Blot Analysis

The small RNA subfraction was extracted from *E*. *coli* using a small RNA purification kit (Sureprep Small RNA purification kit, Fisher Bio reagents) according to the manufacturer’s instructions. The RNA was resolved by electrophoresis through an 8% urea-polyacrylamide gel in TBE buffer (0.1 M Tris, 0.1 M Borate, 1 mM EDTA, pH 9), blotted to a nylon membrane (Zeta probe GT genomic tested blotting membrane) in TBE buffer at 10 V (room temperature for 1 h). The membrane was UV-cross-linked (120 mJ/cm^2^, UV Stratalinker, Stratagene). ^32^P-labeled oligonucleotides with sequences complementary to the control RNA, NEO1A or NEO4A aptamers were used as probes. The northern blot protocol is described in more detail in Supplementary Information.

## Results

### Aptamers increase intracellular total drug concentration

**Figure 1:**
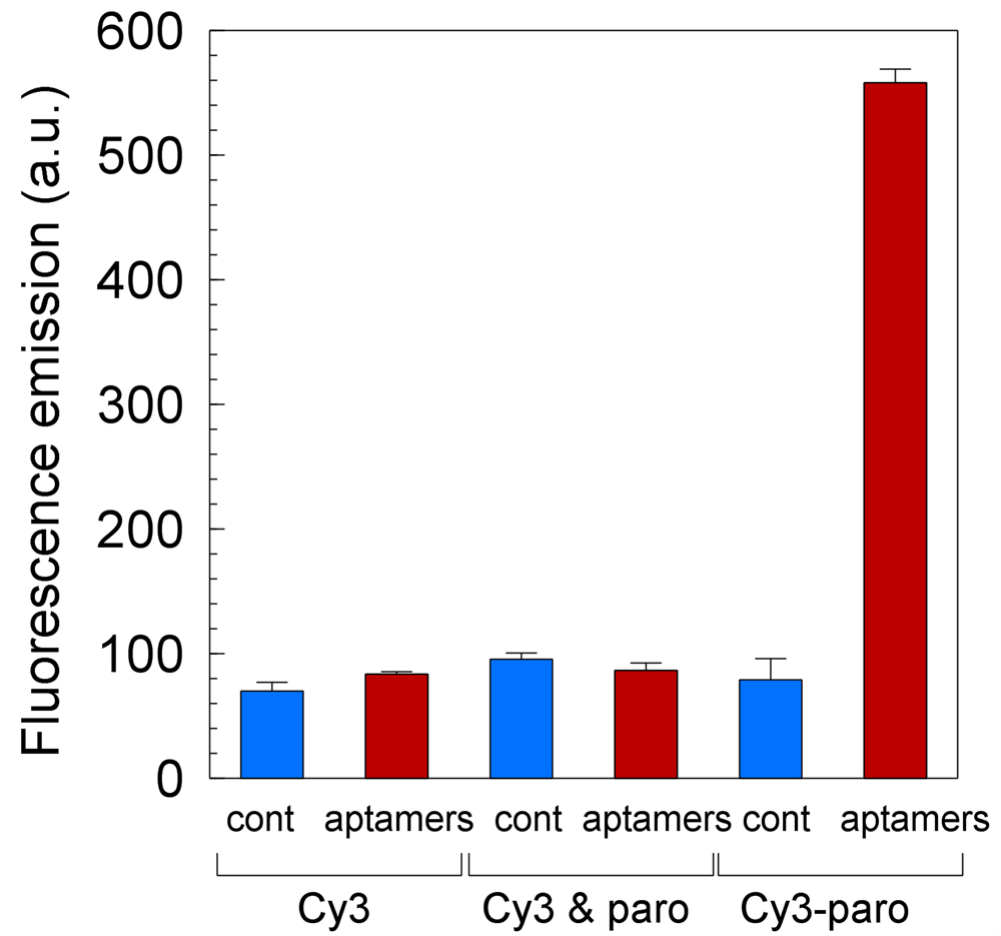
Aptamer expression increases the intracellular concentration of its ligand. *E*. *coli* BL21 cells expressing control RNA (blue bars) or the neomycin-B aptamer, NEO1A (red bars), were grown for three hours in the presence of either 7 μM Cy3, 7 μM Cy3 and 7 μM paromomycin, or 7 μM Cy3-paromomycin, then collected and read by fluorescence spectroscopy with λ_ex_ = 510 nm λ_em_ = 563 nm. The fluorescence for each condition was determined immediately after adding the fluorophores for the 0-time control. This value was subtracted from the fluorescence for the same condition that was obtained after 3 h incubation to obtain the results shown. This result represents a single experiment that was performed in triplicate and is supported by similar results from 7 other independently performed experiments (Figs S1–S3).

To test the hypothesis that mobile aptamers inside a cell could increase the intracellular concentration of a small molecule ligand for which the membrane restricts entry^16^ we used NEO1A, a neomycin-B binding aptamer^18^, and paromomycin labeled with Cy3 as its ligand^19^. The affinity of NEO1A for Cy3-paromomycin was determined by isothermal titration calorimetry (ITC) to be 3.6 ± 0.4 μM when tested in a buffer that approximates the intracellular salt concentrations of mammalian cells^19^. *E*. *coli* expressing the neomycin-B aptamer accumulated Cy3-paromomycin to significantly higher levels than when the same cells expressed a control RNA (Figs 1 and S1–3). Expression of the control and aptamer RNAs were confirmed by northern blot analysis. Cy3 was not accumulated by cells when supplied alone or in combination with paromomycin. These results show that cells expressing aptamers accumulate more ligand than without aptamer expression, but the form of the ligand, e.g. bound to aptamer or free, in the cytosol cannot be distinguished.

### Aptamers increase intracellular free drug concentration

The previous mathematical model predicted that mobile aptamers in the cell will act as DRAGINs and increase the intracellular concentration of the free form of the aptamer ligand. The DRAGIN effect described by the model is due to the aptamer competing for ligand export through the cell membrane and establishing a semi-equilibrium within the cytoplasm that increases the intracellular concentration of the free form of the ligand. Although it is consistent with this prediction, the observed increase in total aptamer ligand could also be accomplished by the aptamer providing additional holding capacity for its ligand, which should lower the intracellular concentration of the free ligand form^16^.

**Figure 2:**
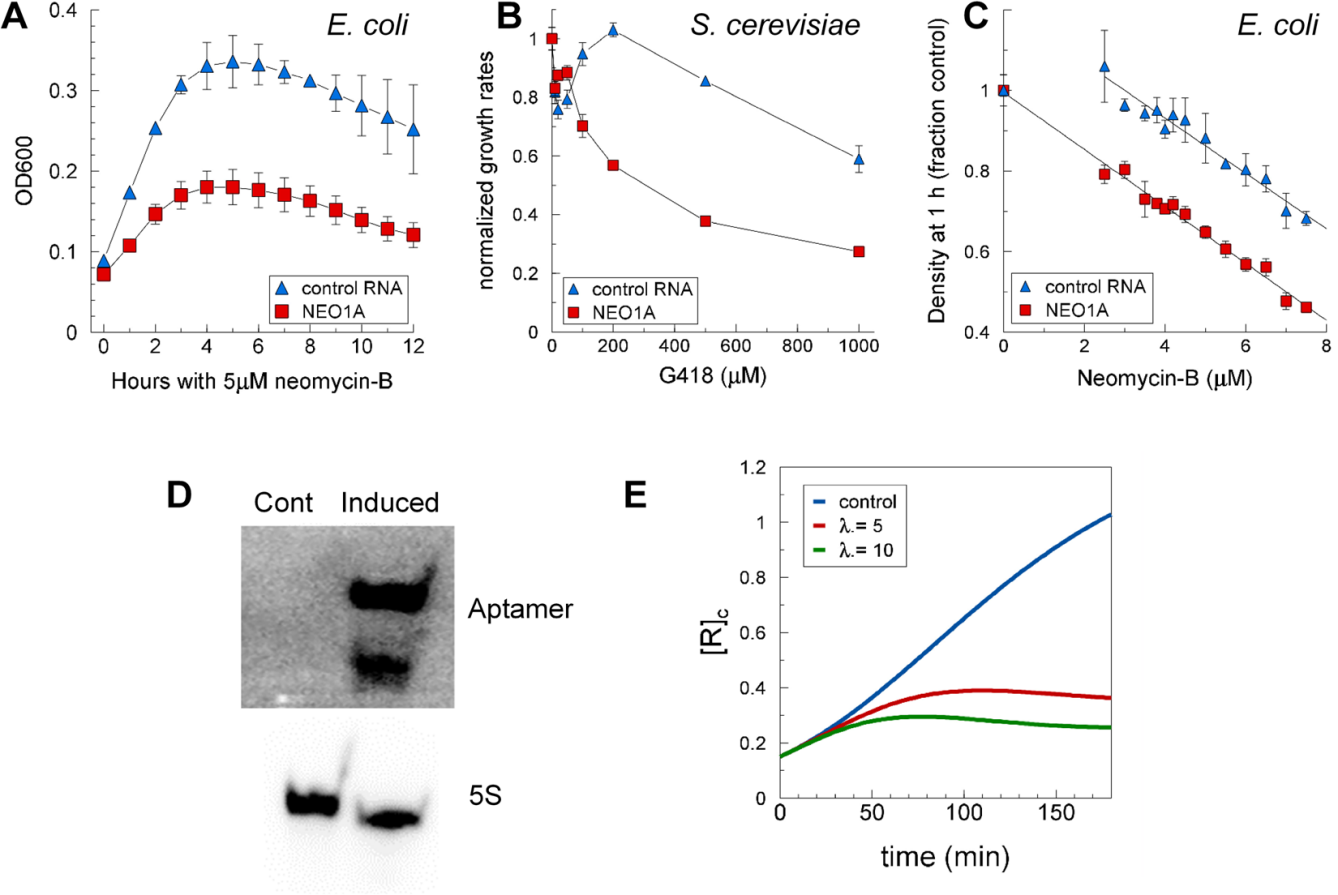
The NEO1A aptamer increases the effectiveness of its ligand in killing cells. (**A**) BL21 cells expressing control RNA or the neomycin-B aptamer, NEO1A, were grown for 8 h after the addition of 1 mM IPTG with their density determined each hour. Error bars are the standard deviations of triplicates. (**B**,**C**) Aptamer expression decreases the IC50 for cell growth in yeast and bacteria: (**B**) *Saccharomyces cerevisiae* (strain BY4735) expressing control RNA (the Spinach aptamer) or NEO1A were grown in SD-Uracil medium with 2% glucose in the presence of various concentrations of G418 and their normalized growth rates were plotted against the G418 concentration. Error bars are equal to 1 minus the coefficient of determination of the regression line for each determined slope. Error bars are associated with each point, but not observed when they are smaller than the height of the symbol. (**C**) *E*. *coli* expressing control RNA or the NEO1A were grown in triplicate in the presence of various concentrations of neomycin-B and cell densities after 1 h were plotted against the neomycin-B concentration with the standard deviations as error bars. This data is representative of three experiments from which similar results were obtained. (**D**) Northern blots of RNA isolated from cells that were induced or not induced by IPTG to produce the NEO1A aptamers for an incubation period of 1 h in the presence of 1 mM IPTG. (**E**) Cellular growth curves predicted by the math model for controls (no aptamer) and cells that produce aptamer at two different rates (λ = 5 and 10), all in the presence of 5 μM inhibitor. This is Fig. 1a in the Math Model (Supplementary Information).

To determine if the intracellular free aminoglycoside concentrations could be raised by the expression of aptamers, we tested the ability of aminoglycosides to kill *E*. *coli* that expressed aminoglycoside aptamers compared with control RNA. If the aptamers were capable of behaving as DRAGINS by increasing the intracellular free ligand concentration, cells expressing aptamers would be killed more effectively than cells expressing control RNAs. However, if the expressed aptamers sequestered the aminoglycosides by increasing holding capacity, the cells were expected to be protected from killing. Consistent with the proposed DRAGIN activity, cells that expressed aptamers grew more slowly in the presence of the same aminoglycoside concentration compared to cells that expressed control RNAs (Fig. 2). This result was demonstrated with several combinations of aptamers and aminoglycosides in bacteria (Figs 2A and S4–S7) and yeast (Fig. 2B). The impact of aptamer expression was to decrease the effective concentration of aminoglycoside required to kill the cells (IC_50_) by 2-3-fold (Fig. 2C). These results were confirmed by minimal inhibitory concentration (MIC) analysis (Fig. S8, Tables S4 and S5). Northern blots of RNA isolated from *E*. *coli* that carried either the control RNA expression plasmid or the aptamer expression plasmids verified that RNAs containing the aptamers were expressed and present in the latter cells and not in the former (Fig. 2D), whereas the control RNA was expressed in the former (Fig. S9).

### A compartment model for the effect of aptamer expression on ligand accumulation in cells

**Figure 3:**
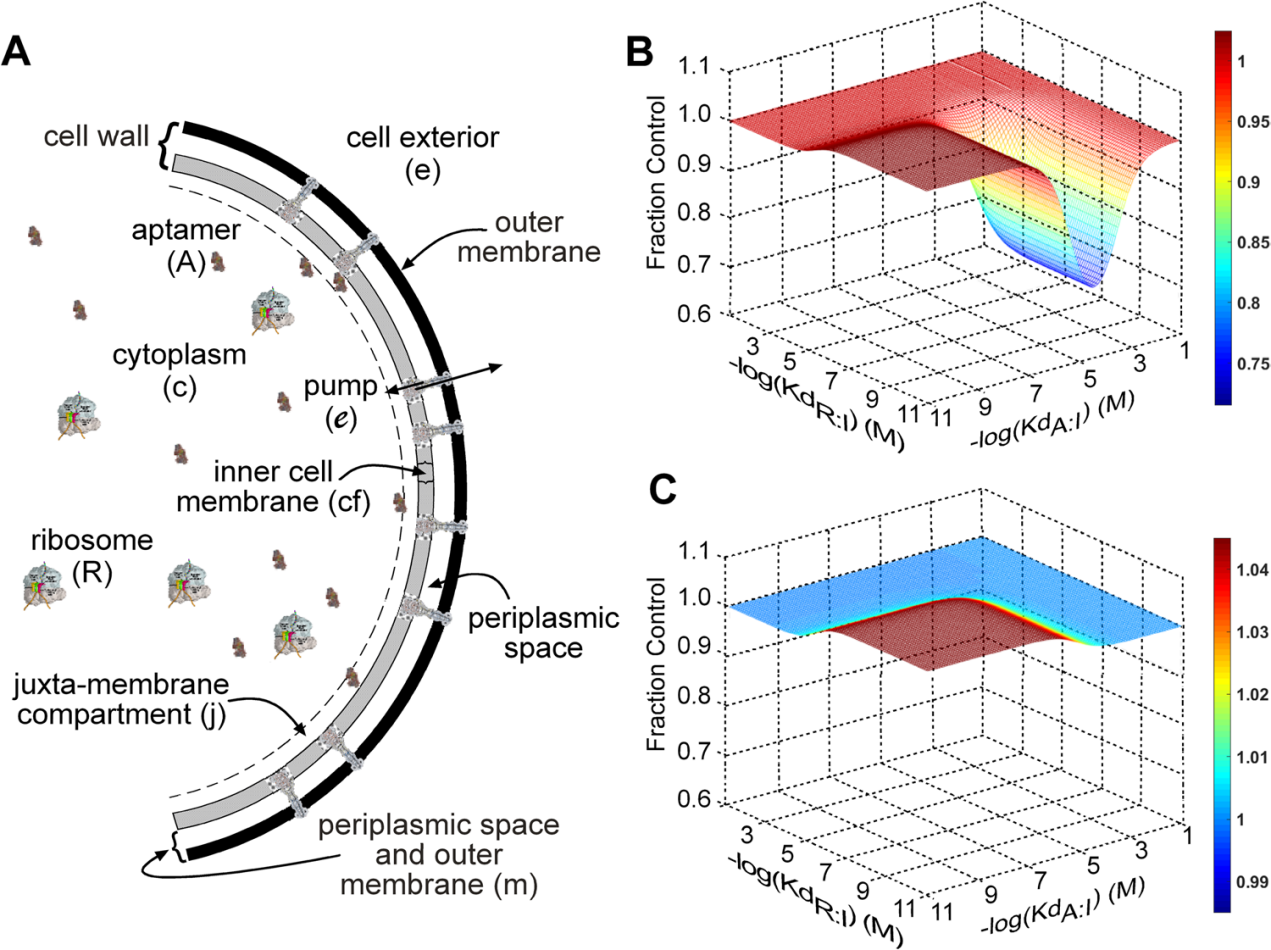
A compartmental model for the effect of expressed aptamers on cell growth. (**A**) The model (see the Math Model (Supplementary Information) for a full mathematical discussion) includes five compartments: (1) cytosol, (2) juxtamembrane space, (3) inner membrane, (4) periplasmic space and outer membrane and (5) cell exterior. The ribosome (R) is the target of the aminoglycosides used in this study. The ribosome (R) to cell ratio is assumed as constant and the ribosome concentration is used as a surrogate for cell density. R has access to the cytosol, but not the juxtamembrane compartment. The aptamers (**A**) expressed by the cells can access both cytosol and juxtamembrane compartment. Pumps capable of exporting the aptamer ligand extend from the inner membrane to the cell exterior. The aptamer ligand (I) is sourced in the extracellular space and is maintained at a constant level. (**B**) Model prediction when the aptamer has high permeability into the juxtamembrane compartment (Fig. 5D, Mathematical model, Supplementary Material). The relation between ribosome affinity and aptamer affinities, expressed as −log(K_d_R:I) and −log (K_d_A:I), and the cell density after one hour, expressed as fraction of control (no ligand/inhibitor). The K_d_s for both aptamer and ribosome were varied by changing the off-rate (k^−1^). (**C**) Model prediction when aptamer has low permeability to the juxtamembrane compartment (Math Model, Supplementary Information). The numerical values used to generate (**B**,**C**) are found in Supplementary Information (Fig. 5A, Math Model, Table 1 or in the caption for Fig. 5).

To evaluate the mechanism by which aptamers increase intracellular ligand concentrations, we turned to a mathematical model. Our previous PDE-based model was replaced with a simplified ordinary differential equation (ODE)-based compartment model (Math Model, Supplementary Information) in which the ribosomes (R), which are the targets of the aminoglycosides, are the proxy for the cells and their proliferation. The model includes five compartments, which are the i) e, cell exterior, ii) m, outer cell membrane and the periplasmic space, iii) cf, inner cell membrane, iv) j, cytoplasmic juxtamembrane space and v) c, remainder of the cytoplasm (Fig. 3A). Ligand concentrations are computed for the cytoplasm, the inner cell membrane and the cell exterior by numerical solutions of the system of ODEs described in the Math Model (Supplementary Information). There are no dynamical equations for the concentrations of the chemical species in the juxtamembrane compartment and the region between the inner membrane and the exterior because those concentrations are related to their counterparts in the ODE regions by the partition coefficients.

Molecular diffusion was a parameter in the PDE model^16^. Entities, such as the ribosome, with relatively low diffusion rates would less frequently encounter the inner membrane surface compared with the smaller more rapidly moving aptamers. In the compartment model, the juxtamembrane space, which is considered to be a thin zone immediately adjacent to the inner membrane surface, is a surrogate for the consequence of a combination of molecular mobility and folded molecular shape and size, which will influence the frequency that a particular molecule will access the inner membrane surface with sufficient intimacy to pick up a ligand molecule. Due to their large size, the ribosome targets of the aminoglycosides are considered to reside in the cytosol with no access to the juxtamembrane space. The smaller aptamers can populate both the cytoplasm and the juxtamembrane space. Drug exporters are present in the membrane/cell wall compartment, and the aminoglycoside ligand is initially added as a bolus to the extracellular space. Because the extracellular volume is very large compared to the cell volume, the extracellular concentration of aminoglycoside is considered a constant.

As did the PDE model, this model predicted that the presence of aminoglycoside-binding aptamers in the cytoplasm would increase the intracellular free aminoglycoside concentration and thereby decrease the extracellular concentration of aminoglycoside required to kill the cells (Fig. 2E), which was what we observed experimentally (Fig. 2A–C). When tested over a range of affinities for aminoglycosides of ribosomes and aptamers, the model predicts a “sweet spot” of combinations of affinities of the aptamer and the ligand target (here the ribosome) in which the experimentally observed DRAGIN activity can occur (Fig. 3). The model predicted that the DRAGIN effect requires access of the aptamer to the juxtamembrane space (cf. Fig. 3B,C). It also showed that aptamers with relatively low ligand affinities (K_d_s in the micromolar to millimolar range) were effective DRAGINS and increased the intracellular concentration of free ligand. Aptamers with very high affinities were predicted to protect the cells due to the aptamer:ligand equilibrium being shifted more towards the aptamer-ligand complex (cf. Fig. 3B,C).

### The DRAGIN effect is dictated by the specificity of the aptamer

**Figure 4:**
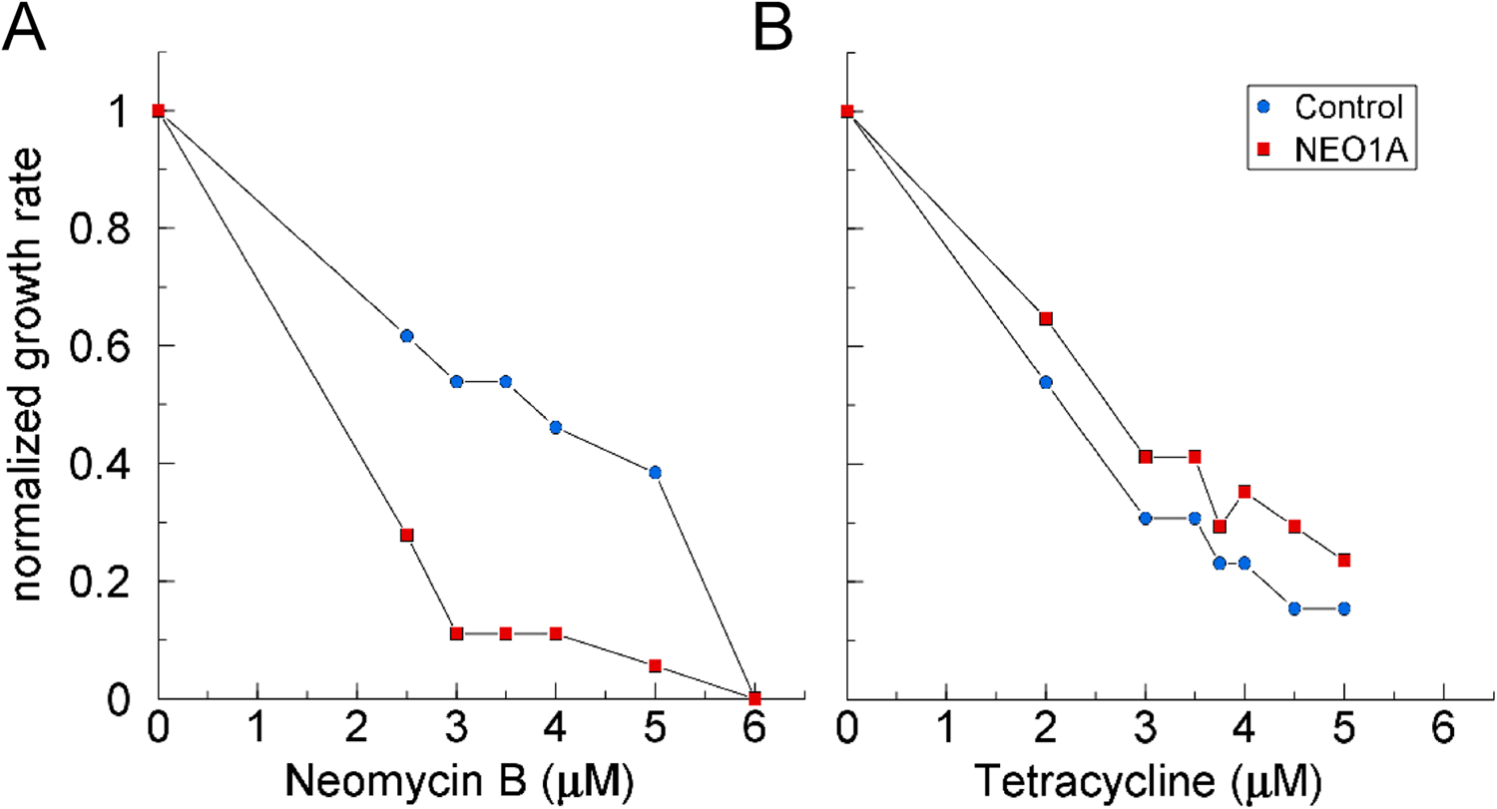
Specificity of the DRAGINs effect for the cognate ligand. BL21 cells expressing the control RNA or the NEO1A aptamer were incubated with various concentrations of neomycin-B (**A**) or tetracycline (**B**) and cultured for 12 h. The maximum growth rates were determined, normalized to the rate for the same cell culture in the absence of drug and plotted as a function of drug concentration. Errors (1-coefficient of determination) for the regression analysis were all smaller than the sizes of the symbols.

If the DRAGIN effect is due to the aptamer interacting with its ligand then it should reflect the ligand specificity of the expressed aptamer. We tested this question with the NEO1A aptamer expressed in cells that were grown in the presence of neomycin-B (Fig. 4A) or tetracycline (Fig. 4B). The expression of NEO1A sensitized the cells to the cognate ligand, neomycin-B, but not to the orthologous ligand, tetracycline. The affinity of NEO1A for tetracycline was so low as to be unmeasurable by ITC (Fig. S10). These results demonstrate that the DRAGINs activity is a function of the specificity of the aptamer expressed by the cells and not due to a nonspecific effect caused by the plasmid presence or IPTG-driven RNA expression. In other experiments, IPTG induction was found to have some effect on the growth rate, particularly at lower inoculum densities (Fig. S11). However, with the same inoculum density and IPTG concentrations, there was no significant difference between the growth rates of cells transformed by expression vectors for the control RNA or the aptamers (Fig. S12).

### Aptamers without DRAGIN activity

**Figure 5:**
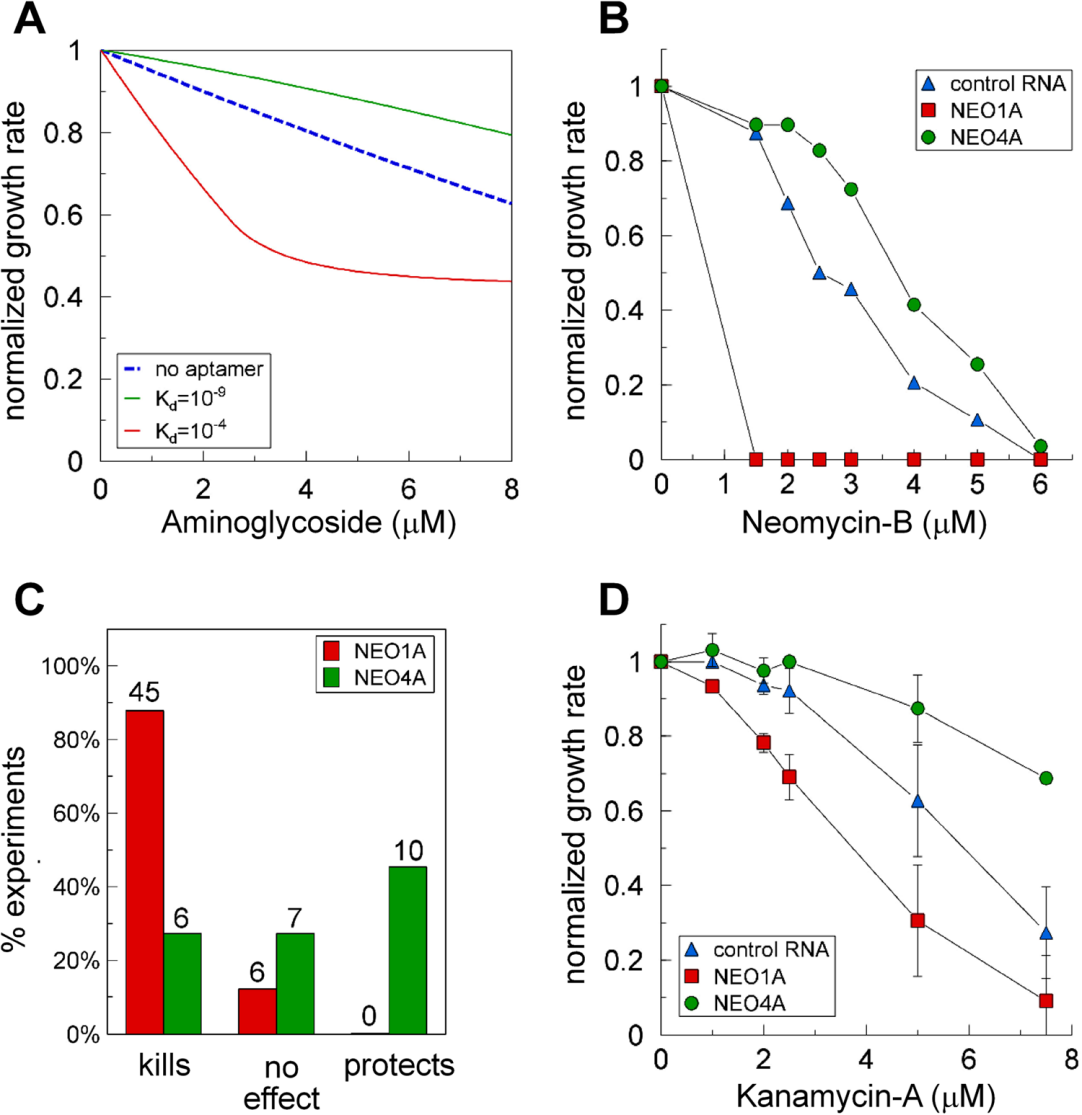
Comparison of two aptamers with different propensities to protect or kill the cells in which they are expressed. (**A**) The math model predictions for the effect on growth rate of cells expressing high affinity (green) or low affinity (red) aptamers (Math Model, Supplementary Information, Fig. 2B). The numerical values used to generate (**A**) are in the Supplementary Information (Math Model, Table 1 and the caption for Fig. 2). (**B**) BL21 cells expressing control RNA, the low affinity NEO1A, or the higher affinity neomycin-B aptamer, NEO4A, were grown in the presence of various concentrations of neomycin-B after induction with 1 mM IPTG. Growth rates were normalized to the initial cell density in each data set. Error bars are smaller than the symbol size. (**C**) The percent of experiments showing killing, protection or no effect by neomycin-B for cells expressing NEO1A and NEO4A compared with cells expressing the control RNA. The numbers over the bars identify the number of independent experiments. (**D**) BL21 cells expressing control RNA, NEO1A, or NEO4A, were grown in the presence of various concentrations of kanamycin-A after induction with 1 mM IPTG. Growth rates were normalized to the initial cell density in each data set. The plot shows an average of the data from 4 experiments, each performed in triplicate.

The compartment model predicted that aptamers with high affinities (K_d_ < 10^−7^ M) would decrease the intracellular concentration of free ligand and protect the cells from killing by a toxic ligand, whereas aptamers with affinities in the “sweet spot” (K_d_s between 10^−3^ and 10^−5^ M) would increase cell killing (Fig. 5A). Although the intracellular K_d_s for these aptamers in the context of the expressed RNAs are not known, the K_d_s measured *in vitro* for these aminoglycoside aptamers are between these two optima. We tested the effect of two neomycin-B aptamers with different affinities for aminoglycosides. The higher affinity aptamer, NEO4A, binds neomycin-B with a K_d_ of 99 nM, whereas the lower affinity aptamer, NEO1A, binds neomycin-B with a K_d_ of 292 nM (Table S5). Consistent with the model prediction, expression of the higher affinity aptamer (NEO4A) frequently protected cells in which it was expressed from killing by neomycin-B. By contrast, expression of the lower affinity aptamer (NEO1A) promoted killing and never protected cells from killing by neomycin-B of the cells in which it was expressed (Fig. 5B). However, the difference in affinities for neomycin-B of NEO1A and NEO4A was only three-fold when measured *in vitro*. With the math model predicting a large affinity range between the optimum for protection vs. killing, it seemed unlikely that this small difference in affinities of these two aptamers would be sufficient to switch an aptamer from a DRAGIN that promotes killing to one that reduces the free intracellular ligand concentration, presumably by sequestering the ligand, to promote protection.

We further investigated the data for NEO1A and NEO4A to establish if the difference between the effects of these two aptamers on killing by neomycin-B is statistically significant. Of a total of 51 independent experiments performed by 5 people (independent operators), NEO1A never promoted protection of the cells in which it was expressed, whereas it promoted killing by neomycin-B in 88% (45) of these experiments (Fig. 5C). In some instances, several ligands were compared in the same experiments for the effect of aptamer expression on killing compared with control RNA expressed by a parallel cell culture. In total, of 114 separate tests of the effect of NEO1A expression (as a singlemer or multimer) on killing by a variety of ligands, NEO1A mostly promoted killing (70%) and protection against killing was not observed with any of the aminoglycoside ligands. By contrast, the distribution of effects on killing due to the expression of NEO4A was quite different: Of 23 independently performed experiments by 3 people, only 6 showed the promotion of killing and 10 promoted protection (Fig. 5C). For all ligands the numbers for NEO4A expression were kills (6), no effect (7) and protects (15). Statistical analysis of the data represented in Fig. 5C using Fisher’s exact test for independence of the distribution between the three effects (kill, no effect, protection) for the two aptamers yielded a p value of 3.4 × 10^−8^, which rejects the null hypothesis and supports our conclusion that NEO1A promotes killing and NEO4A tends to promote protection. The variable protective response of NEO4A could be due to this aptamer not having quite a high enough affinity for the ligand to always protect or not always being expressed at high enough levels to achieve protection.

To further test if the function (protection or killing) of these aptamers is due to their affinities for their ligands, we tested the ability of the NEO1A and NEO4A aptamers to protect from or enhance cell killing by a ligand for which both had much lower affinity than for neomycin-B. Kanamycin-A, has affinities for NEO1A and NEO4A of 43 and 11 µM respectively. Similar to the results for neomycin-B, NEO4A protected the cells from killing by kanamycin-A in two of two experiments, whereas NEO1A promoted cell killing by kanamycin-A in all 14 experiments in which it was tested (Fig. 5D). Thus, although the relative affinities of the aptamers for kanamycin-A were the same as for neomycin-B, with NEO4A having the higher affinity in each case, it is unlikely that the relative ligand affinities of these aptamers are primarily responsible for the DRAGIN or protection effects on cell growth rate. This suggests that another parameter must be important in determining the effect of a particular aptamer on the intracellular concentration of its ligand in the cells in which it is expressed.

### Impact of access to the juxtamembrane space for DRAGIN activity

**Figure 6:**
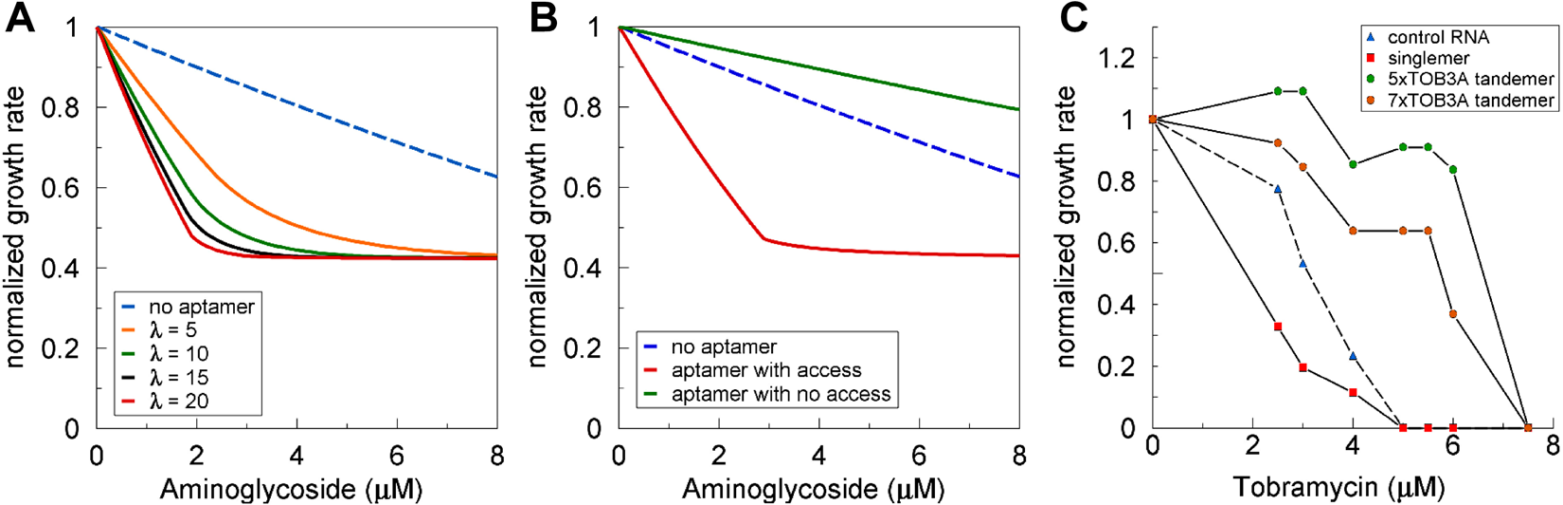
Tobramycin aptamers enhance lethality as singlemers and protect from killing when in tandem arrays. (**A**) The expression level of aptamers (Kd = 10^−4^ M) is varied in the compartment model (Fig. 3A, Math Model, Supplementary Information). (**B**) Access to the juxtamembrane compartment is severely restricted in the compartment model (Fig. 6B, Math Model, Supplementary Information). The numerical values used to generate. (**A**,**B**) are to be found in the Supplementary Information (Math Model, Table 1 and the captions for Figs 3 and 6). (**C**) Cells expressing control RNA, singlemers, and tandemers of five and seven tobramycin aptamers were tested for their growth rates in the absence or presence of tobramycin from 2.5 to 7.5 μM. Errors (1-coefficients of determination) are smaller than the symbol sizes.

To investigate other possible contributors to the killing or protective activity of aminoglycoside aptamers expressed in cells, we first modeled the effect of changing the level of expression of the aptamer. At higher concentrations in the cell, we speculated that the aptamer might become a “sponge” for the ligand. However, the model showed that increasing the source rate of an aptamer that promotes killing increased the killing activity and did not result in protection from killing by the ligand (Fig. 6A). The highest aptamer concentrations tested in the model were 20 times the concentration of ribosomes. This concentration is much higher than can be expected for an expressed RNA even from a plasmid in bacteria in which the ribosomes are present at about 10^4^ per cell^20^ and all other RNAs combined are present at about 1-2 × 10^3^ per cell^21^.

We evaluated the impact of access to the juxtamembrane compartment. The original PDE model clearly demonstrated that mobility of the aptamer is required for aptamers to increase the intracellular free concentration of drug^16^. The compartment model mimics the original model by including a juxtamembrane compartment that the aptamer can populate, but that is not accessible to larger molecules such as the ribosome target of the aptamer ligand (Fig. 3B, Math Model, Supplementary Information). Without access to the juxtamembrane space, the aptamer cannot increase the intracellular concentration of its ligand and promote killing (Figs 3B and 5B). Thus, DRAGIN activity might not be observed with an aptamer that is linked to a larger molecule that would be less mobile and have less direct access to the internal membrane surface. We tested this hypothesis using strings of five and seven tobramycin aptamers (TOB3A) that have been previously demonstrated to bind their tobramycin ligands *in vivo*^17,19^. The string of five tandem tobramycin aptamers binds tobramycin with a range of K_d_s between 4.3 and 46 μM compared with the 240 nM K_d_ estimated for the singlemer TOB3A aptamer^19^. According to the model, if affinity plays a predominant role in determining the DRAGIN effect of these aptamers, both singlemer TOB3A and tandemers of 5 or more TOB3A aptamers should either promote cell killing or not have any effect on cell survival. However, singlemers and tandemers differ in size and shape, which could limit molecular access to the inner membrane surface (juxtamembrane space in the model, Math Model, Supplementary Information). Indeed we observed that strings of five and seven TOB3A tandem aptamers protected the cells from killing, while the TOB3A singlemer promoted cell killing by tobramycin (Fig. 6C). The reason for this difference in the effect of singlemer and tandemers on drug efficacy is unlikely to be simply due to the different number of aptamers in tandem in each RNA or the sizes of the RNAs because other data with different aptamers and ligands show the DRAGIN effect with singlemers, 2mers and 3mers (Figs S4–S7). Mobility is a function of the distance cubed and is unlikely to be sufficiently different for the singlemer and 5-tandemer to have contributed to the observed effects. We speculate that the most probable explanation for these results is that the multi-aptamer tobramycin RNAs form 3D structures that limit their access to the juxtamembrane space, with the result that they are ineffective as DRAGINs and effective in protection because they can still bind and sequester some of the ligand that makes its way into the cytosol.

## Discussion

We have shown that intracellular receptors, such as aptamers, can behave as DRAGINs to influence accumulation in the cell of the small molecule(s) that they selectively recognize. The outcome is an increase in the intracellular concentration of the free form of the small molecule ligand when the aptamer is present in the cytosol. The previous PDE-based mathematical model predicted that the DRAGIN activity would be observed for aptamers with affinities for their ligands in a certain range, which encompasses the affinities reported for most aptamers^16^. With the compartmental model we show that, at sufficiently high levels of expression, aptamers with high affinities could protect the cells by capturing and retaining the incoming ligand. Here and in a previous report^22^, some aptamers were found to protect the cells in which they were expressed from killing by their drug ligand. In our studies, a comparison of the effects of NEO1A and NEO4A that bind neomycin-B with different affinities showed that the lower affinity NEO1A reliably demonstrated DRAGIN activity, whereas the higher affinity NEO4A aptamer had a significant tendency towards cell protection. However, these same tendencies were demonstrated for NEO1A and NEO4A with the ligand kanamycin-A for which both have lower affinities than for neomycin-B. We also showed that RNA size cannot be correlated with demonstrated DRAGIN activity and speculate that the 3D folding of the RNAs containing the aptamer sequences might be a critical feature for determining if an intracellularly expressed aptamer is a DRAGIN. Consistent with this postulate, we have found that, whereas NEO4A protects cells when expressed from a Trc promoter, it has no effect when expressed from T7 promoter, which is also regulated by LacO. Northern blots showed that RNAs of different sizes were produced from these promoters, which might fold differently in the cytosol (Fig. S9).

The current compartment model and the previous PDE model^16^ included features that allowed access of the aptamers to the inner surface of the cell membrane. Intimate membrane access was allowed in the PDE model by aptamer mobility and in the ODE compartment model by access to the juxtamembrane compartment. Thus, we speculate that an important property of a DRAGIN aptamer is its ability to intimately access the inner surface of the membrane and pick off aminoglycosides hovering at the inner membrane face that would otherwise be removed by diffusion through the membrane or by export enabled by drug pumps. For example, an aptamer may be more likely to be a DRAGIN if the ligand binding pocket can closely approach the inner surface of the membrane. Our observations that the TOB3A aptamer is a DRAGIN as a singlemer and protects as a tandemer and that the NEO4A aptamer DRAGIN properties depend on its RNA sequence context are consistent with the possibility that RNA structure might play a role in determining if an aptamer can perform as a DRAGIN. It is also likely that features of the drug will be important for enabling DRAGIN activity. For example, aminoglycosides are positively charged and so might hover for a longer time on the inner membrane surface than would a drug that is hydrophobic or not positively charged. This longer dwell time may provide more opportunity for an aptamer to pick off its ligand and transport it into the cytosol.

An important insight from these studies is that aptamers with DRAGIN activity can increase the available intracellular concentrations of their ligands. Thus, DRAGIN aptamers might provide a means of expanding drug treatments to some currently unreachable drug targets. In this vein, another means of breaking the undruggable barrier was identified with the previous PDE model by which it was demonstrated that, if the target enzyme was made mobile, it performed the same DRAGIN function as the aptamer^16^. However, as for aptamers, protein structure and the location of the drug binding site on the protein target along with chemical properties of the drug may play a role in enabling capture of the drug in the juxtamembrane region.

RNAs are proposed to have performed the work of replication in primitive cells prior to the advent of proteins. Early life is proposed to have first consisted of RNA polymers capable of replication, which may have first been created under conditions of repeated concentration due to cycles of drying and rehydration that promoted polymerization over hydrolysis^23^. Eventually these polymers became surrounded by a membrane, which first may have consisted of ethers and fatty acids and later evolved to consist of phospholipids or glycerophosphate ethers after the split of the *Bacteria* and *Archaea* domains^2^. The hydrophobic primitive membrane would have been a barrier for precursors of RNA synthesis. Although precursor entry can be enabled by flipping of individual lipid components of the membrane, a primitive cell would have been advantaged for evolution if it were able to more rapidly and selectively capture and concentrate precursors for RNA synthesis. Our results suggest that RNA aptamers could have performed this function by selectively picking off the appropriate precursors from the primitive membrane and moving them away from the inner membrane surface from which they might otherwise have been flipped again to the outside. In this regard, it is of interest that a relatively large percentage of known riboswitches (9 of 20) recognize molecules structurally related to nucleosides. Comparison of the TPP riboswitch sequences in eubacteria and archaebacteria resulted in this riboswitch being described as a “molecular fossil” with an evolutionary origin that predates the appearance of proteins and the transcriptional and translational events that it regulates in modern cells^15^. Considering that central metabolism of modern cells is driven by the intracellular concentrations of small molecules that are brought in from the extracellular environment, some RNAs in modern cells may contain aptamer-like domains with DRAGIN activities that influence the accumulation and availability of certain nutrients brought in by membrane-localized protein transporters.

In our studies we have used RNA aptamers as the intracellular receptors to shift the distribution of their ligands into the cell. However, the math model does not define the molecular entity. Thus, small mobile proteins, or other polymers with specificity for an identified target drug, might also perform the DRAGIN function. The development of molecules with a DRAGIN function might have several modern-day applications. In addition to improving chemotherapeutics to reach currently undruggable targets, another potential area of application of DRAGINs is in synthetic biology to promote the intracellular accumulation of selected small molecules. Microbes might be developed that express DRAGINs for environmental toxins to promote their intracellular accumulation for environmental cleansing. Aptamers expressed from specific gene promoters could also be used to image cells with radiolabeled nontoxic aptamer ligands.

In summary, our results demonstrate that small RNA aptamers can selectively increase the intracellular free concentrations of their respective ligands. Mathematical modeling identified ligand affinity as one parameter that determines DRAGIN activity, but with the limited range of aptamer affinities available for study, experimentation found little correlation of ligand affinity with DRAGIN activity. This suggests that other features are also required in the model of which we speculate that the most important is the ability of the aptamer to closely approach the intracellular surface of the plasma membrane, which likely depends on RNA structure. Although RNA structure cannot be incorporated into a compartment model, accessibility of the ligand binding site for the inner membrane can be modeled by altering access to the juxtamembrane space. This proxy for RNA structure produced results from the model consistent with the hypothesis that RNA structure might be an important parameter that determines DRAGIN activity. Our findings support a feasible hypothesis to explain the means by which primitive cells might have concentrated synthetic precursors. The concept that intracellular entities can influence the intracellular accumulation of small molecules coming from the outside adds a new dimension to understanding how cellular metabolism might be driven and how the concentrations of drugs, toxins and energy sources could be altered in cells.

## Electronic supplementary material

Supplementary Information

## Data Availability

All data discussed in this report is available from the authors on request.

## References

[1] Lohse PA, Szostak JW (1996). Ribozyme-catalysed amino-acid transfer reactions. Nature.

[2] Martin W., Russell M. J. (2003). On the origins of cells: a hypothesis for the evolutionary transitions from abiotic geochemistry to chemoautotrophic prokaryotes, and from prokaryotes to nucleated cells. Philosophical Transactions of the Royal Society B: Biological Sciences.

[3] Ralser M (2014). The RNA world and the origin of metabolic enzymes. Biochem Soc Trans.

[4] Hanczyc MM, Fujikawa SM, Szostak JW (2003). Experimental models of primitive cellular compartments: encapsulation, growth, and division. Science.

[5] Xavier JC, Patil KR, Rocha I (2014). Systems Biology Perspectives on Minimal and Simpler Cells. Microbiology and Molecular Biology Reviews: MMBR.

[6] Chen IA, Salehi-Ashtiani K, Szostak JW (2005). RNA Catalysis in Model Protocell Vesicles. Journal of the American Chemical Society.

[7] Schrum JP, Zhu TF, Szostak JW (2010). The Origins of Cellular Life. Cold Spring Harbor Perspectives in Biology.

[8] Monnard P-A, Deamer DW (2002). Membrane self-assembly processes: Steps toward the first cellular life. The Anatomical Record.

[9] Wei C, Pohorille A (2014). Flip-Flop of Oleic Acid in a Phospholipid Membrane: Rate and Mechanism. The Journal of Physical Chemistry B.

[10] Budin I, Szostak JW (2011). Physical effects underlying the transition from primitive to modern cell membranes. Proc Natl Acad Sci USA.

[11] Tuerk C, Gold L (1990). Systematic evolution of ligands by exponential enrichment: RNA ligands to bacteriophage T4 DNA polymerase. Science.

[12] Ellington AD, Szostak JW (1990). *In vitro* selection of RNA molecules that bind specific ligands. Nature.

[13] Tuerk C (1997). Using the SELEX combinatorial chemistry process to find high affinity nucleic acid ligands to target molecules. Methods Mol Biol.

[14] Wang, T., Cong, X. & Nilsen-Hamilton, M. In *Making and Using Antibodies* (eds Howard, G. C. & Kaser, M. R.) Ch. 8, 173–206 (Taylor & Francis Group, 2013).

[15] Winkler W, Nahvi A, Breaker RR (2002). Thiamine derivatives bind messenger RNAs directly to regulate bacterial gene expression. Nature.

[16] Boushaba K, Levine HA, Nilsen-Hamilton M (2009). A mathematical feasibility argument for the use of aptamers in chemotherapy and imaging. Math Biosci.

[17] Ray J (2016). IMAGEtags: Quantifying mRNA Transcription in Real Time with Multiaptamer Reporters. Methods Enzymol.

[18] Wang Y, Killian J, Hamasaki K, Rando RR (1996). RNA molecules that specifically and stoichiometrically bind aminoglycoside antibiotics with high affinities. Biochemistry.

[19] Shin I (2014). Live-cell imaging of Pol II promoter activity to monitor gene expression with RNA IMAGEtag reporters. Nucleic Acids Res.

[20] Golding I, Cox EC (2006). Physical Nature of Bacterial Cytoplasm. Physical Review Letters.

[21] Moran MA (2012). Sizing up metatranscriptomics. The Isme Journal.

[22] Werstuck G, Green MR (1998). Controlling gene expression in living cells through small molecule-RNA interactions. Science.

[23] Ross DS, Deamer D (2016). Dry/Wet Cycling and the Thermodynamics and Kinetics of Prebiotic Polymer Synthesis. Life.

