# Supplementary material for: Aptamer-enabled uptake of small molecule ligands
Source: Sci Rep. 2018 Oct 24;8:15712. doi: 10.1038/s41598-018-33887-w (PMC6200808; doi:10.1038/s41598-018-33887-w)
Supplement: Supplementary file 1 — Supplementary Information [file 41598_2018_33887_MOESM1_ESM.pdf]

Aptamer-enabled uptake of small molecule ligands

Supplemental Materials

Supipi Liyamali Auwardt[1,2,3], Yeon-Jung Seo[1], Muslum Ilgu[1,2,3,4], Judhajeet Ray[1,2,5], Robert R. Feldges[2], Shambhavi Shubham[1,2,6], Lee Bendickson[1,2], Howard A. Levine[1], and Marit Nilsen-Hamilton[1,2,3*]

[1]Iowa State University, Ames, IA, [2]Ames National Laboratory, Ames IA, [3]Aptalogic Inc., Ames IA,

* To whom correspondence should be addressed.

Tel: 515-294-9996; Fax: 515-294-0453; marit@iastate.edu

Present Address:

[4]Middle East Technical University, Ankara, Turkey, [5]Cornell University, Ithaca, NY, [6]Integrated DNA Technologies, Coralville IA

Table of Contents

Northern blot analysis 3

Table S1. Expected transcripts produced from plasmids encoding aptamers used in this study 4

Table S2. Expected transcripts produced from plasmids encoding control RNAs and natural RNA fragments used in this study 5

Table S3. Expected transcripts produced from plasmids encoding tandem aminoglycoside-binding RNAs used in this study 6

Table S4. Minimal inhibitory concentration (MIC) of neomycin-B is lower in cells that express NEO1A aptamers. 7

Table S5. Control conditions for minimal inhibitory concentration (MIC) studies 7

Table S6. Dissociation constants of the RNAs used in this study for neomycin and kanamycin-class aminoglycosides 8

Table S7. Dissociation constants for NEO1A and NEO4A aptamers for their aminoglycoside ligands 8

Figure S1. Qualitative evaluation of the effect of aptamer expression on accumulation of Cy3-paromomycin by *E. coli* BL21 cells 9

Figure S2. Emission spectra of extracts from *E. coli* BL21 cells that expressed control RNA or aptamer incubation with Cy3-paromomycin 10

Figure S3. Quantitative evaluation of the effect of aptamer expression on accumulation of Cy3-paromomycin by E. coli BL21 cells 11

Figure S4. Effect of expression of NEO1A on bacterial growth rates in the presence of kanamycin-A. 12

Figure S5. Effect of the expression of a variety of aminoglycoside binding RNAs on bacterial growth in the presence of kanamycin-B. 13

Figure S6. Comparison of bacterial growth with and without natural RNA elements that bind aminoglycosides. 14

Figure S7. The effect of expressing natural RNA elements that bind aminoglycosides on bacterial growth rates. 15

Figure S8. Minimal inhibitory concentration (MIC) for neomycin-B 16

Figure S9. Northern blot of RNAs extracted from BL21 cells that expressed control RNA or NEO4A. 17

Figure S10. ITC thermograms for the binding interactions of NEO1A with neomycin-B and tetracycline. 18

Figure S11. Effect of IPTG on cell growth at two inoculum densities. 19

Figure S12. Comparison of bacterial growth with and without the aptamers. 20

MATHEMATICAL MODEL 21

A Compartment Model for Aptamer-Enabled Uptake of Small Molecule Ligands, Howard A. Levine & Yeon-Jung Seo 21

METHODS

Northern blot analysis

BL21 (DE3) star *E. coli* (Invitrogen, Eugene, OR) were transformed with the bacterial expression vectors and grown for 18h at 37°C in Luria-Bertani broth (LB) with 100μg/mL ampicillin. The cultures were diluted 100 times and again grown at 37°C to OD = 1.0. The cells were again diluted 100 times in LB and grown for 1 h with 1mM IPTG at 37°C. The RNA was extracted from these *E. coli* cells by the Sureprep Small RNA purification kit (Fisher Bio reagents). The extracted RNA was resolved in 8% urea-polyacrylamide gel in TBE buffer. The resolved RNA was transferred to nylon membrane (Zeta probe GT genomic tested blotting membrane) in TBE buffer at 10V (room temperature for 1h). After the transfer, the nylon membrane was UV-cross-linked (120000uJ/cm2, UV Stratalinker, Stratagene). Then the membrane was incubated with 12 mL of hybridization buffer for 6 h at 47°C for blocking and subsequently incubated with a 5' ^{32}P-labeled NEO1A probe (GGACTAAACTTCTCGCCCAGTCC) or NEO4A probe (TAATACGACTCACTATAGGGAGACAAGCCTCCGCTATA) for 16 h at 60°C. The membrane was washed 2 times at 25 ºC in 0.5X SSC buffer, 0.1% SDS for 15 min each time and then washed another 2 times at 47°C in 0.2X SSC buffer, 0.1% SDS for 15 min each time. The membrane was imaged using a phosphorimager (GE Healthcare)m then stripped and incubated with the 5' ^{32}P-labeled control RNA probe (GTTTTATTAAGCTTGTCG) using the same conditions as for the NEO1A or NEO4A hybridization. For a loading control, the membrane was again stripped and reprobed with the 5' ^{32}P-labeled 5S rRNA-specific probe (GCATGGGGTCAGGTGGGACC).

TABLES

Table S1. Expected transcripts produced from plasmids encoding aptamers used in this study

aptamer	plasmid	promoter	Transcript
KNA1A	pMU174<br>K1.3	T7<br>(Bacterial)	GGGUCGA**GCGCAGUGGGCAUAGAACCAUGCGCAGC**UUAAUAAAACGAAAGGCUCA<br>GUCGAAAGACUGGGCCUUUCGUUUUA^UCUGUUGUUUGUCGGUGAACGCUCUCCU<br>GAGUAGGACAAAUCCGCCGGGAGCGGAUUUGAACGUUGCGAAGCAACGGCCCGGA<br>GGGUGGCGGGCAGGACGCCCGCCAUAAACUGCCAGGCAUCAAAUUAAGCAGAAGGC<br>CAUCCUGACGGAUGGCCUUUU
NEO1A	pMU174<br>N1.3	T7<br>(Bacterial)	GGGUCGA**GGACUGGGCGAGAAGUUUAGUCC**AGCUUAAUAAAACGAAAGGCUCAGU<br>CGAAAGACUGGGCCUUUCGUUUUA^UCUGUUGUUUGUCGGUGAACGCUCUCCUGA<br>GUAGGACAAAUCCGCCGGGAGCGGAUUUGAACGUUGCGAAGCAACGGCCCGGAGG<br>GUGGCGGGCAGGACGCCCGCCAUAAACUGCCAGGCAUCAAAUUAAGCAGAAGGCCA<br>UCCUGACGGAUGGCCUUUU
NEO1A	pJJR152	5S rRNA<br>(yeast)	GGUUGCGGCCAUAUCUACCAGAAAGCACCGUUUCCCGUCCGAUCAACUGUAGUUAA<br>GCUGGUAAGAGCCUGACCGAGUAGUGUAGUGGGUGACCAUACGCGAAACUCAGGU<br>GCUGCAAUCUGUCGACGCCCGGAUAGCUCAGUCGGUAGAGCAGCGGCCG**GGACUG**<br>**GGCGAGAAGUUUAGUCC**CGGCCGCGGGUCCAGGGUUCAAGUCCCUGUUCGGGCGC<br>CAUCUAGACGGACUUCGGUCCGCUUUUUUU
NEO4A	pJJR248	T7<br>(Bacterial)	GGGUCGA**CUUUGCGAUGUCCUUUAAUGGUCCGCGAGG**UCGACAAGCUUAAUAAA<br>ACGAAAGGCUCAGUCGAAAGACUGGGCCUUUCGUUUUA^UCUGUUGUUUGUCGGU<br>GAACGCUCUCCUGAGUAGGACAAAUCCGCCGGGAGCGGAUUUGAACGUUGCGAAG<br>CAACGGCCCGGAGGGUGGCGGGCAGGACGCCCGCCAUAAACUGCCAGGCAUCAAAU<br>UAAGCAGAAGGCCAUCCUGACGGAUGGCCUUUU
NEO4A	pJJR267	GAL 1<br>(yeast)	AGUAUCAACAAAAAAUUGUUAAUAUACCUCUAUACUUUAACGUCAAGGAGAAAAAA<br>CCCCGGAUCGGACUACUAGCAGCUGUAAUACGACUCACUAUAGGGAAUAUUAAGCU<br>CAAAAAGGAUCCAAAGUCGACGCCCGGAUAGCUCAGUCGGUAGAGCAGCGGCCG**CU**<br>**UUGCGAUGUCCUUUAAUGGUCCGCGAGG**CGGCCGCGGGUCCAGGGUUCAAGUCCC<br>UGUUCGGGCGCCAUCUAGAGGGCCGCAUCAUGUAAUUAGUUAUGUCACGCUUACA<br>UUCACGCCCUCCCCCCACAUCCGCUCUAACCGAAAAGGAAGGAGUUAGACAACCUGA<br>AGUCUAGGUCCCUAUUUAUUUUUUUAUAGUUAUGUUAGUAUUAAGAACGUUAUU<br>UAUAUUU
SPN1A<br>(Spinach)	pJJR167	5S rRNA<br>(yeast)	GGUUGCGGCCAUAUCUACCAGAAAGCACCGUUUCCCGUCCGAUCAACUGUAGUUAA<br>GCUGGUAAGAGCCUGACCGAGUAGUGUAGUGGGUGACCAUACGCGAAACUCAGGU<br>GCUGCAAUCUGUCGACGCCCGGAUAGCUCAGUCGGUAGAGCAGCGGCCG**GACGCAA**<br>**CUGAAUGAAAUGGUGAAGGACGGGUCCAGGUGUGGCUGCUUCGGCAGUGCAGCU**<br>**UGUUGAGUAGAGUGUGAGCUCCGUAACUAGUCGCGUC**CGGCCGCGGGUCCAGGG<br>UUCAAGUCCCUGUUCGGGCGCCAUCUAGACGGACUUCGGUCCGCUUUUUUU

Legend: Possible sequences for the RNA transcripts containing aptamers are shown with red carats to identify potential early termination sites created during the cloning. The aptamer sequences are identified as bolded and underlined. Where there is an optional early termination site, the aptamer sequence is still within the transcribed RNA.

Table S2. Expected transcripts produced from plasmids encoding control RNAs and natural RNA fragments used in this study

RNA	plasmid	promoter	Transcript
control	pJJR269	GAL 1	AGUAUCAACAAAAAAUUGUUAAUAUACCUCUAUACUUUAACGUCAAGGAGAAAAAACCC<br>CGGAUCGGACUACUAGCAGCUGUAAUACGACUCACUAUAGGGAAUAUUAAGCUCAAAAA<br>GGAUCCAAAGUCGACGCCCGGAUAGCUCAGUCGGUAGAGCAGCGGCCGCGGGUCCAGGG<br>UUCAAGUCCCUGUUCGGGCGCCAUCUAGAGGGCCGCAUCAUGUAAUUAGUUAUGUCAC<br>GCUUACAUUCACGCCCUCCCCCCACAUCCGCUCUAACCGAAAAGGAAGGAGUUAGACAAC<br>CUGAAGUCUAGGUCCCUAUUUAUUUUUUUAUAGUUAUGUUAGUAUUAAGAACGUUAU<br>UUAUAUUU
control	pMU159	T7	GGGUCGACAAGCUUAAUAAAACGAAAGGCUCAGUCGAAAGACUGGGCCUUUCGUUUUA^<br>UCUGUUGUUUGUCGGUGAACGCUCUCCUGAGUAGGACAAAUCCGCCGGGAGCGGAUUU<br>GAACGUUGCGAAGCAACGGCCCGGAGGGUGGCGGGCAGGACGCCCGCCAUAAACUGCCA<br>GGCAUCAAAUUAAGCAGAAGGCCAUCCUGACGGAUGGCCUUUU
RRE	pMU174-R1.5	T7	GGGUCGA**GGUGGGCGCAGCUUCGGCUGACGGUACACC**AGCUU<br>AAUAAAACGAAAGGCUCAGUCGAAAGACUGGGCCUUUCGUUUUA<br>^UCUGUUGUUUGUCGGUGAACGCUCUCCUGAGUAGGACAAAUCC<br>GCCGGGAGCGGAUUUGAACGUUGCGAAGCAACGGCCCGGAGGG<br>UGGCGGGCAGGACGCCCGCCAUAAACUGCCAGGCAUCAAAUUAA<br>GCAGAAGGCCAUCCUGACGGAUGGCCUUUU
16S	pMU174-D1.5	T7	GGGUCGA**GGCGUCACACCUUCGGGUGAAGUCGCC**AGCUUAAUA<br>AAACGAAAGGCUCAGUCGAAAGACUGGGCCUUUCGUUUUA^UCU<br>GUUGUUUGUCGGUGAACGCUCUCCUGAGUAGGACAAAUCCGCC<br>GGGAGCGGAUUUGAACGUUGCGAAGCAACGGCCCGGAGGGUGG<br>CGGGCAGGACGCCCGCCAUAAACUGCCAGGCAUCAAAUUAAGCA<br>GAAGGCCAUCCUGACGGAUGGCCUUUU
TSM	pMU174-S1.3	T7	GGGUCGA**CCCCCCCGCCGCGCCAUGCCUGUGGCCGGUCGG**AG<br>CUUAAUAAAACGAAAGGCUCAGUCGAAAGACUGGGCCUUUCGUU<br>UUA^UCUGUUGUUUGUCGGUGAACGCUCUCCUGAGUAGGACAAA<br>UCCGCCGGGAGCGGAUUUGAACGUUGCGAAGCAACGGCCCGGA<br>GGGUGGCGGGCAGGACGCCCGCCAUAAACUGCCAGGCAUCAAA<br>UUAAGCAGAAGGCCAUCCUGACGGAUGGCCUUUU

Legend: Possible sequences for the RNA transcripts containing aptamers are shown with red carats to identify potential early termination sites created during the cloning. The RNA sequences are identified as bolded and underlined. Where there is an optional early termination site, the relevant RNA sequence is still within the transcribed RNA.

Table S3. Expected transcripts produced from plasmids encoding tandem aminoglycoside-binding RNAs used in this study

RNA	plasmid	promoter	Transcript
NEO1A<br>3mer	pMU174-N3.3	T7	GGGUCGA**GGACUGGGCGAGAAGUUUAGUCCAAAAGGACUGGGCGAGAAGUU**<br>**UAGUCCAAAAGGACUGGGCGAGAAGUUUAGUCC**AGCUUAAUAAAACGAAAGG<br>CUCAGUCGAAAGACUGGGCCUUUCGUUUUA^UCUGUUGUUUGUCGGUGAACG<br>CUCUCCUGAGUAGGACAAAUCCGCCGGGAGCGGAUUUGAACGUUGCGAAGCAA<br>CGGCCCGGAGGGUGGCGGGCAGGACGCCCGCCAUAAACUGCCAGGCAUCAAAU<br>UAAGCAGAAGGCCAUCCUGACGGAUGGCCUUUU
RRE<br>2mer	pMU190-R3.1	T7	GGGUCGA**GGUGGGCGCAGCUUCGGCUGACGGUACACCAAAAGGUGGGCGCAG**<br>**CUUCGGCUGACGGUACACC**AGCUUAAUAAAACGAAAGGCUCAGUCGAAAGACU<br>GGGCCUUUCGUUUUA^UCUGUUGUUUGUCGGUGAACGCUCUCCUGAGUAGGA<br>CAAAUCCGCCGGGAGCGGAUUUGAACGUUGCGAAGCAACGGCCCGGAGGGUGG<br>CGGGCAGGACGCCCGCCAUAAACUGCCAGGCAUCAAAUUAAGCAGAAGGCCAUC<br>CUGACGGAUGGCCUUUU
TOB3A<br>5mer	pMU174-T5.3	T7	GGGUCGACGGUCUCAUGCCAAAA**GGCACGAGGUUUAGCUACAC**<br>**UCGUGCCAAAAGGCACGAGGUUUAGCUACACUCGUGCCAAAAG**<br>**GCACGAGGUUUAGCUACACUCGUGCCAAAAGGCACGAGGUUUA**<br>**GCUACACUCGUGCCAAAAGGCACGAGGUUUAGCUACACUCGUG**<br>**CC**AGAGACAAGCUUAAUAAAACGAAAGGCUCAGUCGAAAGACUG<br>GGCCUUUCGUUUUA^UCUGUUGUUUGUCGGUGAACGCUCUCCU<br>GAGUAGGACAAAUCCGCCGGGAGCGGAUUUGAACGUUGCGAAG<br>CAACGGCCCGGAGGGUGGCGGGCAGGACGCCCGCCAUAAACUG<br>CCAGGCAUCAAAUUAAGCAGAAGGCCAUCCUGACGGAUGGCCUU<br>UU
TOB3A<br>7mer	pMU174-T10.3	T7	GGGUCGACGGUCUCAUGCCAAAA**GGCACGAGGUUUAGCUACAC**<br>**UCGUGCCAAAAGGCACGAGGUUUAGCUACACUCGUGCCAAAAG**<br>**GCACGAGGUUUAGCUACACUCGUGCCAAAAGGCACGAGGUUUA**<br>**GCUACACUCGUGCCAAAAGGCACGAGGUUUAGCUACACUCGUG**<br>**CCAAAAGGCACGAGGUUUAGCUACACUCGUGCCAAAAGGCACG**<br>**AGGUUUAGCUACACUCGUGCC**AGAGACAAGCUUAAUAAAACGAA<br>AGGCUCAGUCGAAAGACUGGGCCUUUCGUUUUA^UCUGUUGUUU<br>GUCGGUGAACGCUCUCCUGAGUAGGACAAAUCCGCCGGGAGCG<br>GAUUUGAACGUUGCGAAGCAACGGCCCGGAGGGUGGCGGGCAG<br>GACGCCCGCCAUAAACUGCCAGGCAUCAAAUUAAGCAGAAGGCC<br>AUCCUGACGGAUGGCCUUUU
TSM<br>3mer	pMU190-S3.2	T7	GGGUCGA**CCCCCCCGCCGCGCCAUGCCUGUGGCCGGUCGGAA**<br>**AACCCCCCCGCCGCGCCAUGCCUGUGGCCGGUCGGAAAACCCC**<br>**CCCGCCGCGCCAUGCCUGUGGCCGGUCGG**AGCUUAAUAAAACG<br>AAAGGCUCAGUCGAAAGACUGGGCCUUUCGUUUUA^UCUGUUGU<br>UUGUCGGUGAACGCUCUCCUGAGUAGGACAAAUCCGCCGGGAG<br>CGGAUUUGAACGUUGCGAAGCAACGGCCCGGAGGGUGGCGGGC<br>AGGACGCCCGCCAUAAACUGCCAGGCAUCAAAUUAAGCAGAAGG<br>CCAUCCUGACGGAUGGCCUUUU

Table S4. Minimal inhibitory concentration (MIC) of neomycin-B is lower in cells that express NEO1A aptamers.

Neomycin-B concentration (μg/mL)	Cells expressing control RNA	Cells expressing NEO1A
12.5	No growth	No growth
6.25	No growth	No growth
3.12	No growth	No growth
1.56	Growth	No growth
1.00	Growth	No growth
0.781	Growth	Growth
0.500	Growth	Growth
0.390	Growth	Growth
0.195	Growth	Growth
0.097	Growth	Growth

Legend: BL21 cells expressing the NEO1A aptamer or control RNA were spotted on agar plates containing the identified concentrations of neomycin-B and incubated for 12h at 37°C. The plates were observed for the appearance of colonies as a measure of cell growth.

Table S5. Control conditions for minimal inhibitory concentration (MIC) studies

plate	Neomycin B (1mg/mL)	BL21 cells	resulting growth
1	-	-	No growth
2	+	-	No growth
3	+	+	No growth
4	-	+	growth

Legend: LB-agar plates with or without 1 mg/mL neomycin-B, spotted or not with BL21 *E. coli*, were incubated 12h at 37°C. The plates were observed for appearance of colonies as evidence of cell growth.

Table S6. Dissociation constants of the RNAs used in this study for neomycin and kanamycin-class aminoglycosides

	Dissociation Constants (μM)					
Amino-glycosides	NEO1A aptamer	TOB3A aptamer	KNA2A Aptamer	RRE (Rev-Response Element)	TSM (Thymidylate Synthase mRNA region)	16S (16S rRNA Decoding region)
Neomycin-B	0.29 ± 0.054	2.2 ± 0.5	6.3 ± 0.65	3.54 ± 0.68	0.88 ± 0.11	1.93 ± 0.58
Paromomycin	1.4 ± 0.19	17.2 ± 1.96	9.1 ± 1.0	27 ± 4.95	6.29 ± 3.49	5.31 ± 1.85
Kanamycin-B	0.75 ± 0.26	2.43 ± 1.37	5.26 ± 0.39	8.13 ± 1.41	6.5 ± 3.2	8.9 ± 3.54
Kanamycin-A	43 ± 3.6	73.8 ± 3.96	14.4 ± 2.77	32.2 ± 3.39	10.3 ± 6.65	37.1± 4.38
Tobramycin	4.8 ± 0.95	0.24 ± 0.10	7.0 ± 0.53	4.98 ± 0.75	5.75 ± 1.87	10.3 ± 3.87

Legend: The calculated dissociation constants for the RNAs used in this study were determined by ITC at 25 ºC.

Table S7. Dissociation constants for NEO1A and NEO4A aptamers for their aminoglycoside ligands

	**Ligand**	**Kd (μM)**	**N**
**NEO1A**	Neomycin-B	0.29 ± 0.054	8
	Kanamycin-A	43 ± 3.6	3
	Kanamycin-B	0.75 ± 0.26	5
	Tobramycin	4.8 ± 0.95	3
	Paromomycin	1.4 ± 0.19	5
	Cy3-paromomycin	3.6 ± 0.4	2
	Geneticin	26 ± 9.3	5
**NEO4A**	Neomycin-B	0.099 ± 0.036	3
	Kanamycin-A	10.7 ± 4.2	2

Legend: The dissociation constants for NEO1A and NEO4A aptamer were determined in buffer IC by ITC at 25 ºC and are represented as μM. N shows the number of independent repeats contributing the average estimates.

FIGURES

Figure S1. Qualitative evaluation of the effect of aptamer expression on accumulation of Cy3-paromomycin by *E. coli* BL21 cells

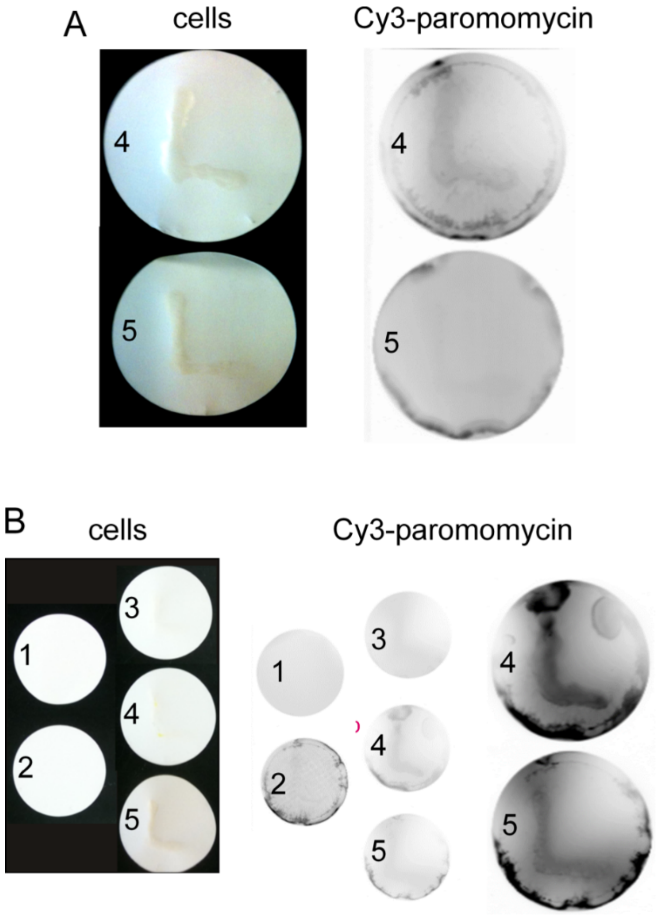

Legend: A) *E. coli* BL21 cells expressing Neo1A aptamers (4) or control RNA (5) were grown for 3 h in the presence of 3 μM of Cy3-paromomcyin, then collected by centrifugation and laid out on a filter in an L shape. The filter was photographed to show the cell population (cells) and imaged for fluorescence using a typhoon scanner (Cy3-paromomycin). **B)** *E. coli* BL21 cells expressing Neo1A aptamers (3, 4) or control RNA (5) were grown for 3 h in the presence of 3 μM of Cy3-paromomycin (4, 5) or in its absence (3), then collected, concentrated, resuspended and laid out on the filter in an L shape then the filters were photographed and imaged as for A). In addition, one filter was imaged without treatment (1) and a second filter was imaged after writing out an L shape with 3 μM of Cy3-paromomycin (3).

Figure S2. Emission spectra of extracts from *E. coli* BL21 cells that expressed control RNA or aptamer incubation with Cy3-paromomycin

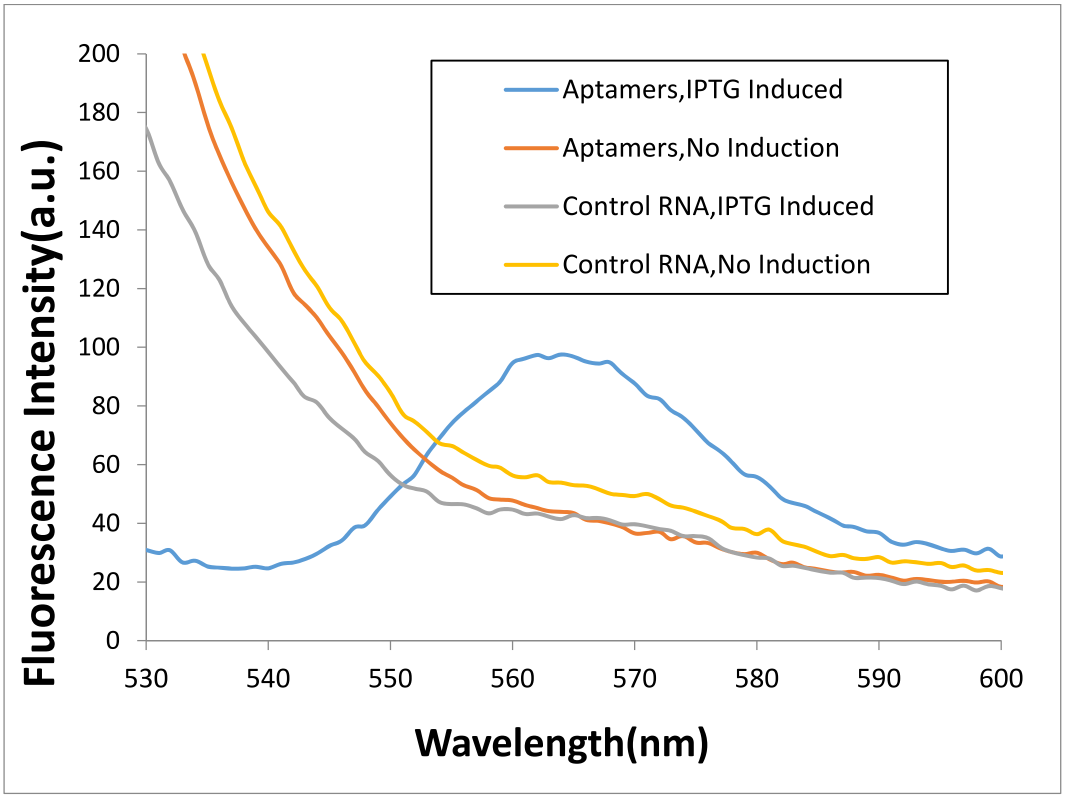

Legend: BL21 cells expressing Neo1A aptamers or control RNA were grown for 3 h in the presence of Cy3-paromomycin with and without induction by 1mM IPTG. The cells were then collected and the fluorescence read by fluorescence spectroscopy with λ_{ex} = 510 nm λ_{em} = 563 nm. Plotted on the Y axis is the residual fluorescence determined after subtracting the fluorescence of the equivalent number of cells that had been incubated briefly (time zero) with Cy3-paromomycin.

Figure S3. Quantitative evaluation of the effect of aptamer expression on accumulation of Cy3-paromomycin by E. coli BL21 cells

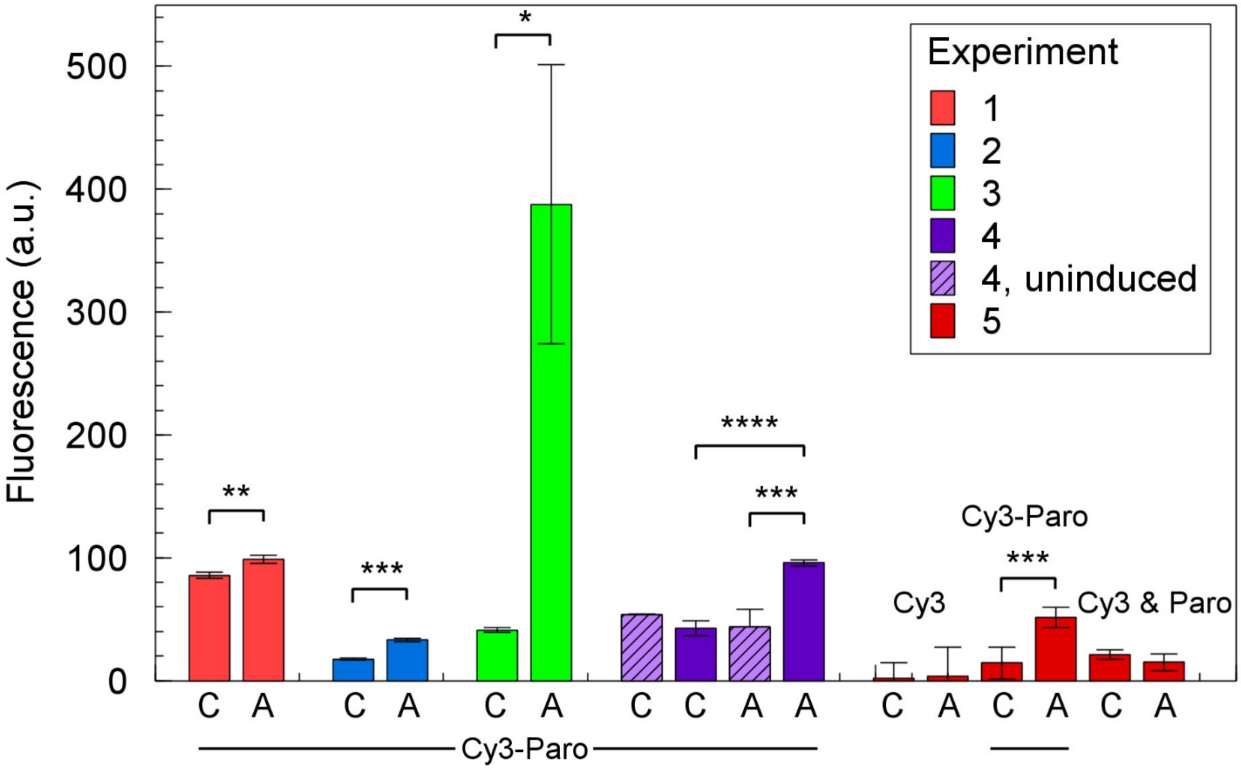

Legend: The results of five independently performed experiments in which cells expressing control RNA (C) or neomycin aptamer-containing RNA (A) were incubated for 0 or 3 h with Cy3-paromomycin (Cy3-Paro) or Cy3 or Cy3 and paromomycin (Cy3 & Paro). The results of each experiment are represented by bars of a different color from other experiments. Cells were induced with 1 mM IPTG for one hour prior to the addition of Cy3-paromomycin, Cy3 or Cy3 and paromomycin (open bars) or were not induced (hashed bars). The concentrations of Cy3 paromomycin used in these experiments were 3 μM (1,5), 5 μM (2) or 7 μM (3,4). Error bars are the standard deviations for duplicate (experiments 1-3) or triplicate independent estimates (experiments 4 and 5). Statistical evaluations for each experiment by Student's t test revealed statistical significance only for the differences between control RNA (C) and aptamer-expressing cells (A) and aptamer expressing cells vs. non-induced cells that had been incubated with Cy3-paramomycin. *, $p<0.05$; **, $p<0.01$; ***, $p<0.005$; ****, $p<0.001$.

Figure S4. Effect of expression of NEO1A on bacterial growth rates in the presence of kanamycin-A.

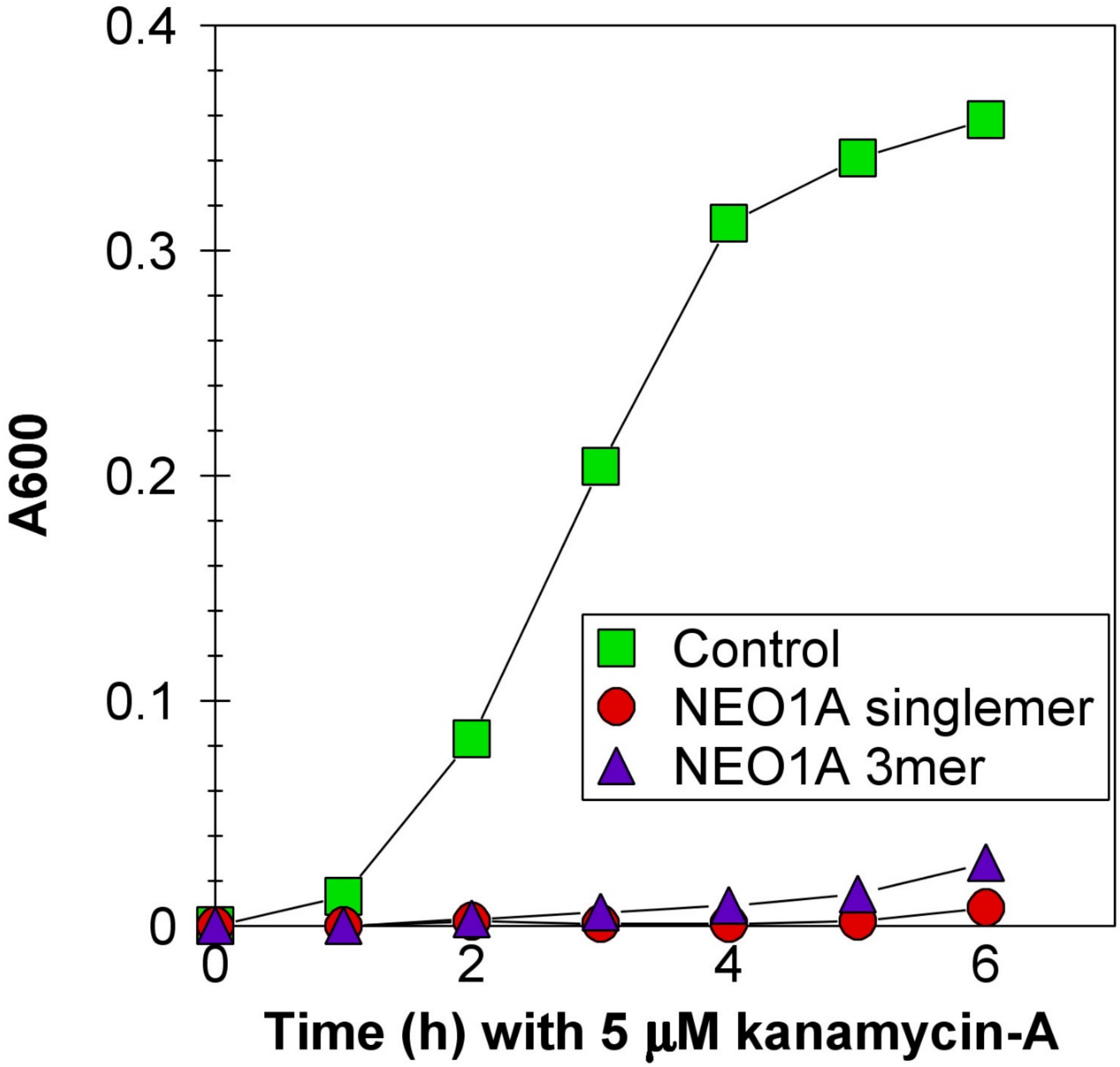

Legend: Aptamer expression decreased the growth of bacteria in the presence of 5 μM kanamycin-A. Control cells with no aptamer expression (green squares). Expression of one unit (red circles) and three tandem units of NEO1A (3mer, purple triangles).

Figure S5. Effect of the expression of a variety of aminoglycoside binding RNAs on bacterial growth in the presence of kanamycin-B.

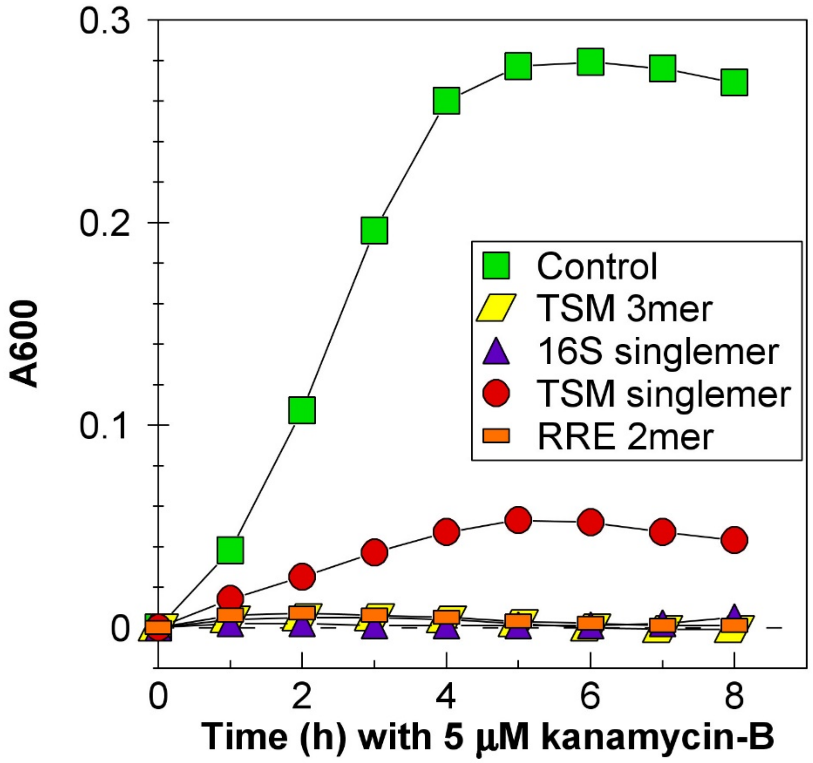

Legend: A range of aminoglycoside binding RNAs were expressed in BL21 cells and tested for their effects on cell growth in the presence of 5 µM kanamycin-B. A single unit of 16S A-site minimal RNA (purple triangles), three tandem units of thymidylate synthase mRNA region (yellow parallelograms), a single unit of thymidylate synthase mRNA region (TSM, red circles), two tandem units of the RRE (orange rectangles), and the control cells with no aptamer expression (green squares).

Figure S6. Comparison of bacterial growth with and without natural RNA elements that bind aminoglycosides.

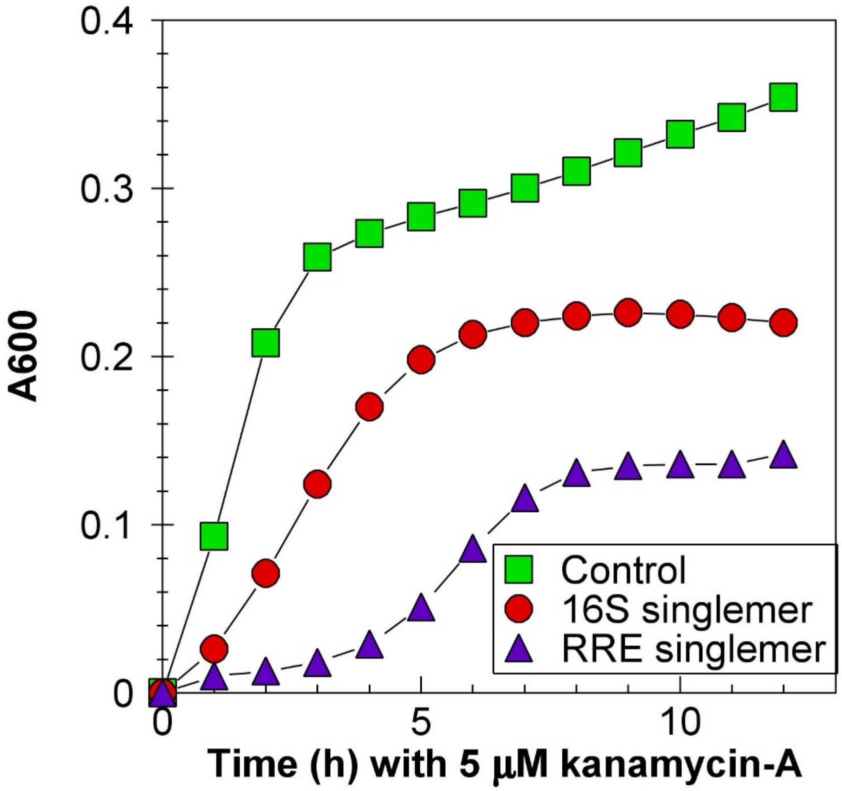

Legend: Expression of single units of aminoglycoside-binding RNA elements from the 16S RNA decoding region or the HIV Rev response element (RRE) decreased the growth rates of bacteria in the presence of 5 μM kanamycin-A compared with control cells. Control cells with no aptamer expression (green squares), 16S A-site minimal RNA (purple triangles), RRE (orange circles).

Figure S7. The effect of expressing natural RNA elements that bind aminoglycosides on bacterial growth rates.

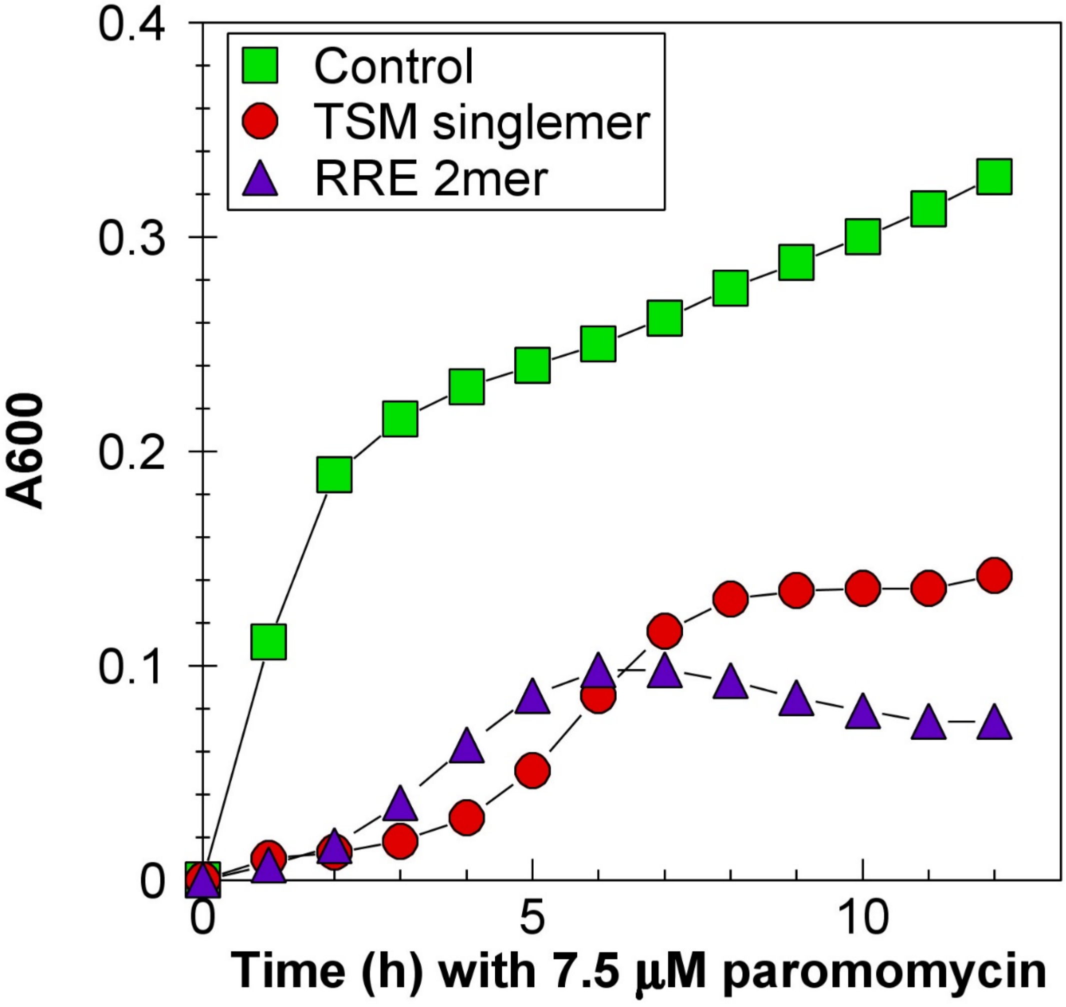

Legend: The growth rates of bacteria expressing natural RNA elements from thymidylate synthase mRNA (TSM) or the HIV Rev response element (RRE) in the presence of 7.5uM paromomycin were compared with control cells not expressing aptamer. Control cells (green squares), single units of TSM (red circles), two tandem units of RRE (purple triangles).

Figure S8. Minimal inhibitory concentration (MIC) for neomycin-B

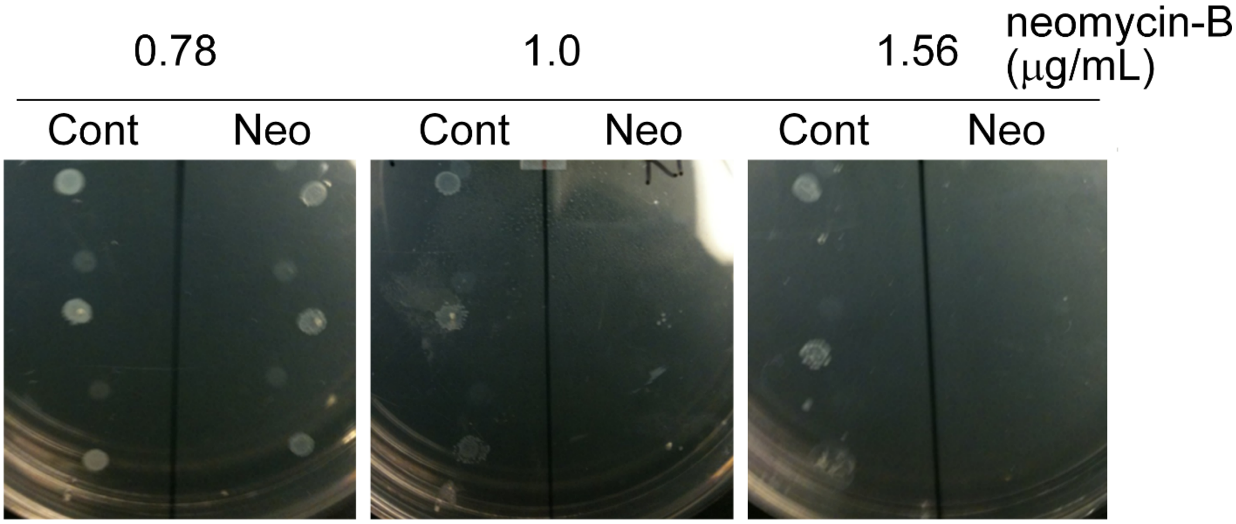

Legend: BL21 cells that expressed the control RNA or the NEO1A aptamer were spotted on agar containing various concentrations of neomycin-B and incubated for 12 h at 37°C. The colony sizes for cells expressing the control RNA (Cont) and NEO1A aptamers (Neo) are shown for three concentrations of neomycin. Table S2 shows the results of all concentrations tested. Similar results were shown in three independent experiments with all showing a decrease in the MIC for neomycin-B for cells that express the NEO1A aptamer.

Figure S9. Northern blot of RNAs extracted from BL21 cells that expressed control RNA or NEO4A.

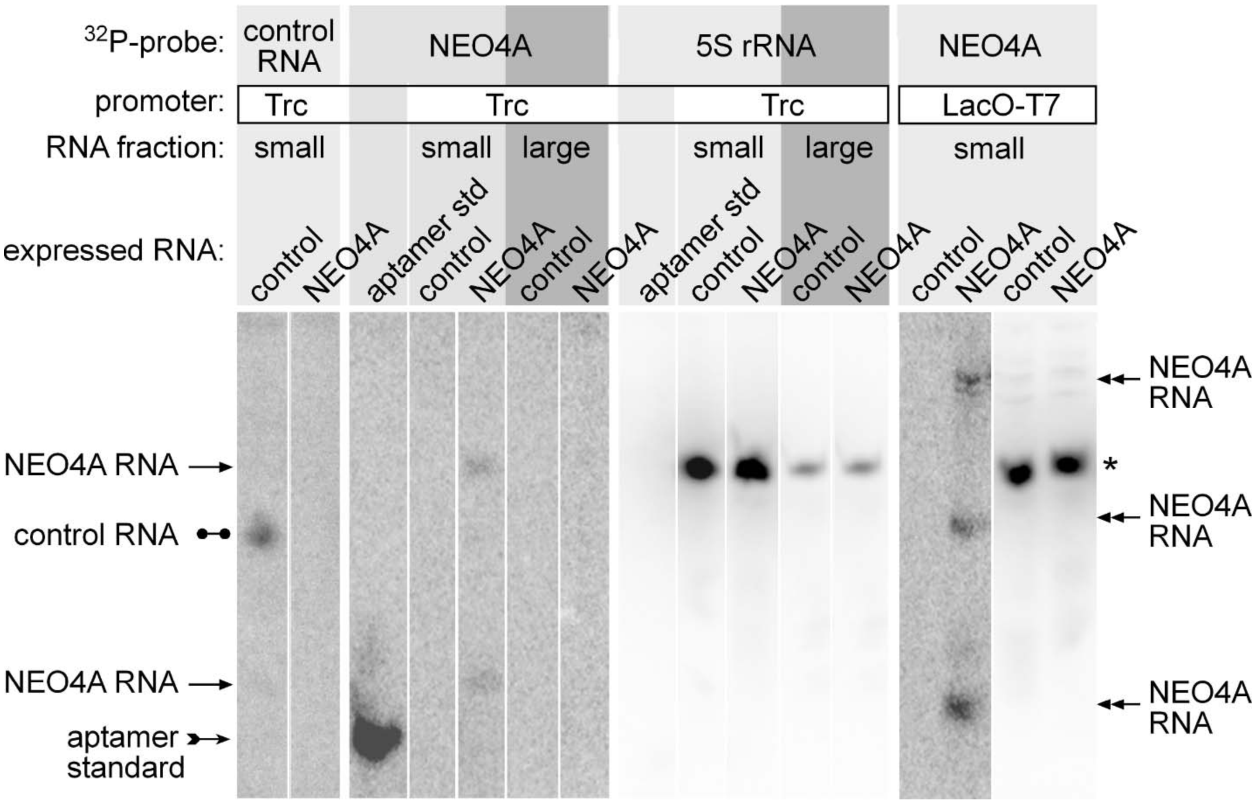

Legend: RNA was extracted from BL21 cells that expressed the control RNA or RNA containing the NEO4A aptamer from the pTrc promoter or the T7 promoter controlled by the lacO operator in response to stimulation by IPTG. The RNA was separated by size into large and small RNA fractions. The northern blot was prepared and blotted with the ^{32}P-labeled complements to control RNA, NEO4A or 5S RNA as indicated. The position of the RNA products are identified with two different sets of NEO4A-containing RNA expressed from the two promoters identified by single arrows (from Trc) or double arrows (from LacO-T7). The position of the 5S RNA is shown by the asterisk.

Figure S10. ITC thermograms for the binding interactions of NEO1A with neomycin-B and tetracycline.

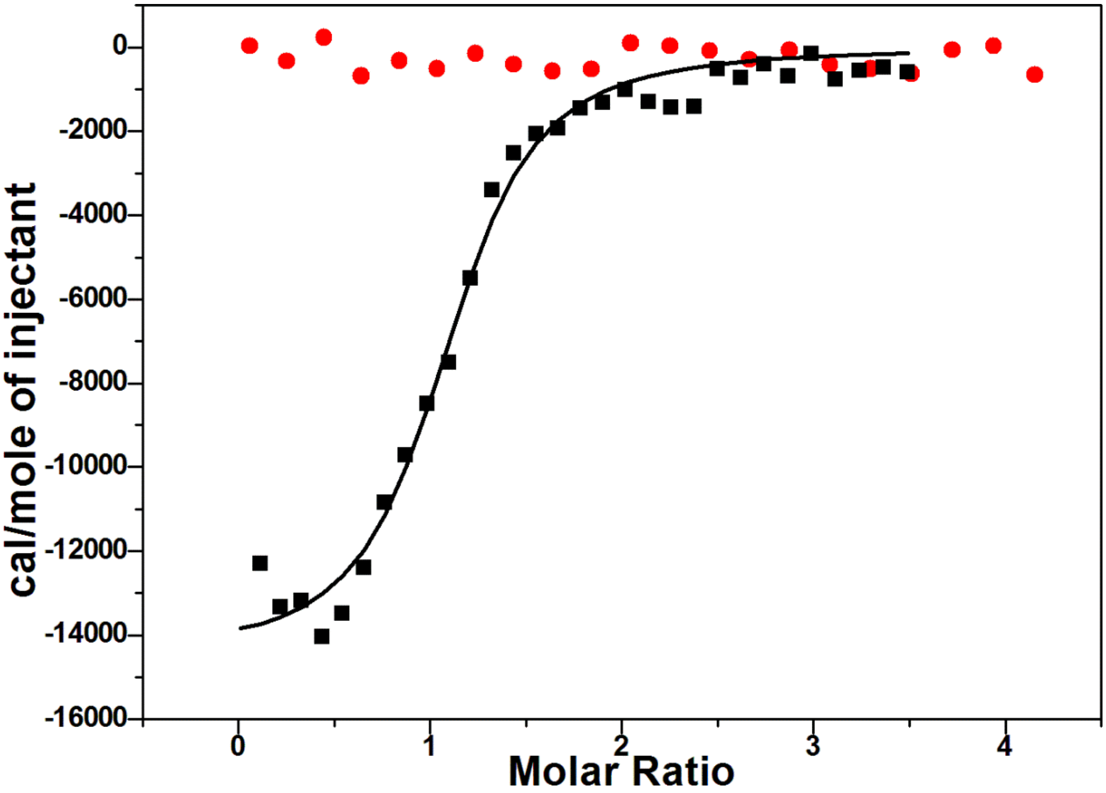

Legend: ITC was used to measure the heat change due to the interaction between NEO1A and tetracycline (red circles) or neomycin-B binding (black squares). The Kd for measured for the interaction of NEO1A and neomycin-B in this experiment was 217 ± 40 nM. Tetracycline showed no evidence of binding the aptamer. The initial concentration of aptamer in the cell was 4 μM for the neomycin titration and 20 μM for the tetracycline titration.

Figure S11. Effect of IPTG on cell growth at two inoculum densities.

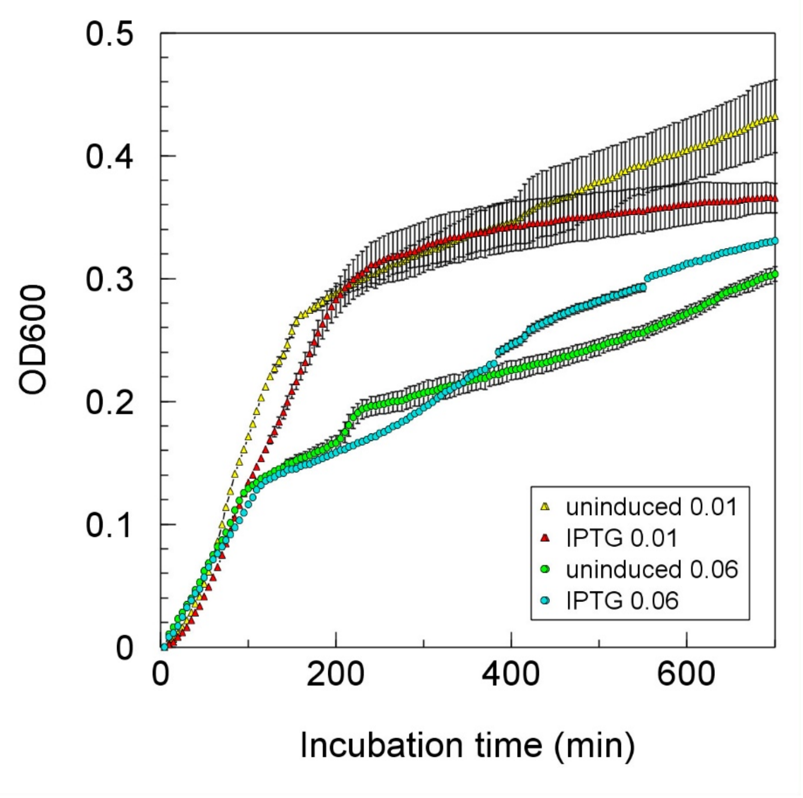

Legend: The growth rates of BL21 expressing NEO1A were inoculated at densities of 0.01 (triangles) and 0.06 (circles) and treated with (red and green) or without (yellow and blue) 1 mM IPTG for 600 minutes over which time the OD600 of each culture was measured each 5 min. The error bars represent the standard deviation for three replicates. All values had the initial inoculum OD600 subtracted. These results are representative of those obtained in three independently performed experiments.

Figure S12. Comparison of bacterial growth with and without the aptamers.

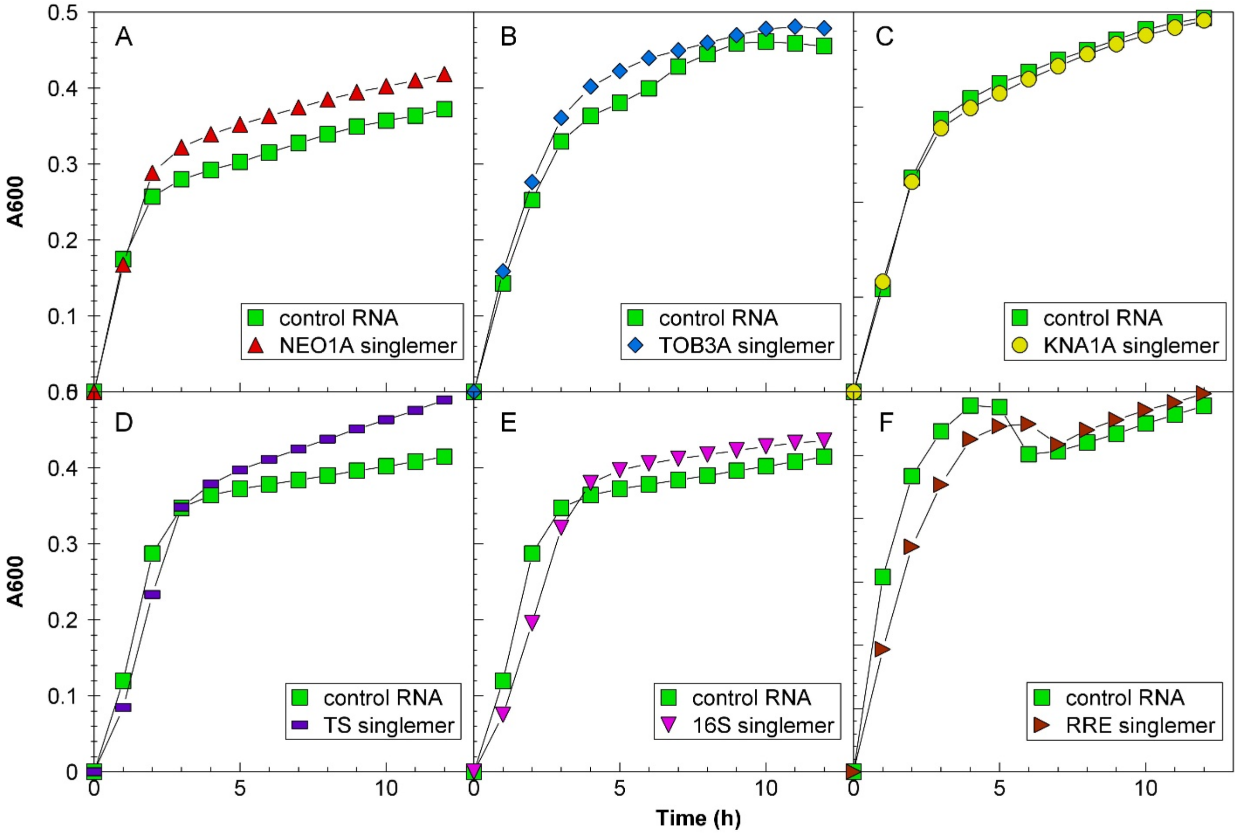

Legend: The growth over 12 h periods of BL21 expressing control RNA (closed squares) are compared with cells expressing **A)** kanamycin aptamer **B)** tobramycin aptamer **C)** neomycin aptamer **D)** thymidylate synthase mRNA region **E)** Rev-response element **F)** 16S minimal A-site. The coefficient of variation is between 5 to 15% in these experiments. Each point is the average of at least three samples.

A COMPARTMENT MODEL FOR APTAMER-ENABLED UPTAKE OF SMALL MOLECULE LIGANDS

HOWARD A. LEVINE & YEON-JUNG SEO

1. Cellular compartment model

The beauty of a compartment model is that it does not specify the geometric location of one compartment relative to another. However, it sometimes necessary to have such a geometric map in mind. In this model, we construct a five compartment model as follows: Consider four concentric spheres of differing radii. They define five regions, the interior of the smallest sphere, the exterior of the largest sphere and three concentric annular spheres. The interior of the smallest sphere is denoted by (c) and contains the cell chromosome. At the end of the day, the dynamics will take place in three of these compartments, namely, the interior of innermost sphere, the exterior region and annular region between the second and third sphere. One can think of a figure generated by these four spheres and these regions as a simplified version as a simplified version of Figure 3A of the paper as we explain next.

The portion of the cytoplasm of the cell containing the chromosome is also where the region where the ribosome active site is presumed to be accessible to any ribosome inhibitor molecule that can reach (c). The region (c) is then referred to as the ribosome accessible portion of the cytoplasm. The remainder of the cytoplasm is a small annular region around (c) called the juxta-membrane, (j). The assumption is that no interaction is possible between the ribosome and the inhibitor in the region (j) because the active site of the ribosome cannot access it. The region exterior to the largest sphere is the cell exterior (e). The remaining two annular regions are to be thought of as follows: The larger annular region (m) is a proxy for the region consisting of both the periplasmic space and the exterior cell membrane in Figure 3A of the paper while the smaller of the two is the inner cell membrane whose interior bounds the the cytoplasm. We call this interior cell membrane the cytoplasmic face (cf) because its interior faces the region (j). We assume that the inhibitor tends to accumulate in this region by passive diffusion across (m) from the cell exterior (e) before being expelled from the cell via a cell pump that prevents much of a build up in (cf).

The goal here is to establish a coupled system of ordinary differential equations among the compartments (c), (cf) and (e) in the concentration variables for the ribosome, the inhibitor, and when present, the aptamer, as well as the products of the inhibitor with the other two species. This will lead to a coupled system of nine ordinary differential equations in nine unknown concentrations.

The concentrations of the ribosome (more precisely, the active cite for the ribosome) and the inhibitor are assumed to be negligible in the juxta-membrane. In the case of the region (m), the inhibitor concentrations (as well as the inhibitor-pump products) can be incorporated into the inhibitor concentrations in the cytoplasm face or the cell exterior via the appropriate partition coefficients. In this sense, we say that the compartments (j), (m) are the separation(passive) compartments for the active compartments (c), (cf) and (e).

At the outset we assume:

A1. The concentration of the ribosome functions is a proxy for the concentration of the cells in the medium. That is the total available ribosome concentration is proportional to the number of cells in the culture.

Date: August 2, 2018.

A2. The sources for ribosome and the aptamer (if any) occupy a very small portion of the region (c) called the chromosome while the inhibitor is initially given as a bolus in the exterior (e).
A3. The chemistry between the ribosome and the inhibiter can only occur in the ribosome active cite accessible region (c).
A4. The inhibitor enters and leaves the region (cf) across the membrane (m) by passive diffusion as well as by a membrane pump. The increase in inhibitor in the cytoplasm is facilitated by passive diffusion through the membrane and by the pump. However the contribution by the pump is small since the pump favors the removal of the inhibitor from the cytoplasm.
A5. The inhibitor can also cross the juxta-membrane from (cf) to (c) via passive diffusion, but the passive diffusion is weaker than what one might expect from the Stokes-Einstein formula. The justification for this is that the inhibitor is highly charged and prefers to remain in the inner membrane boundary region (cf) before being ejected by the pump or passive diffusion to the exterior (e).
A6. The juxta-membrane functions as a separation passive region for the aptamer **and its products**. That is, the ability of the aptamer to cross it to capture inhibitor in (cf) and return the product to (c) is regulated by passive diffusion given by the Stokes-Einstein assumption.
A7. The concentrations of the aptamer and its product with the inhibitor in the juxtamembrane are presumed to have been converted to eliminated by augmentation of their values in the active regions (c), (cf) using the appropriate partition coefficients.

Notation:xx

$$\begin{aligned}
I &= \text{inhibitor such as tobramycin, keomycin, karomomycin, kanamycin-A, etc.} \\
A &= \text{aptamer, 27TOBB3A, 23NEO1A, etc.} \\
R &= \text{ribosome (enzyme) 16S rRNA mimic} \\
\{I:A\}, \{I:R\} &= \text{inhibitor bound aptamer, inhibitor bound ribosome} \\
S_{A,ch}, S_{R,ch} &= \text{cell synthesis rates for aptamer, ribosome} \\
\delta_m &= \text{mean thickness of the cell membrane} \\
\delta_r &= \text{mean thickness of the juxta-membrane}
\end{aligned}$$

The notation for the juxta-membrane thickness, δ_r is taken to remind us that this thickness is ribosome dependent.

If X is one of the species $I, A, R, \{I : A\}, \{I : R\}$ then $[X]_e, [X]_c, [X]_{cf}, [X]_m, [X]_j$ denote the concentration of the species X in that compartment.

2. Ribosome-inhibitor interaction

The ribosome is inactivated by the inhibitor via the simple mechanism:

$$\{R : I\} \underset{k_1}{\overset{k_{-1}}{\rightleftharpoons}} R + I. \tag{1}$$

Here $k - 1, k_{-1}$ denote the on and off rates. (The association constant is $K_a = k_1/k_{-1}$.) The source of the ribosome is the chromosome. The only chemistry for the ribosome and the inhibitor is presumed to be in the cytoplasm. The relevant rate equations are

$$\frac{d[R]_c}{dt} = k_{-1}[\{I : R\}]_c - (k_1[I]_c + \mu_r)[R]_c + S_{R,ch}, \tag{2}$$

$$\frac{d[\{I : R\}]_c}{dt} = k_1[I]_c\ [R]_c - (k_{-1} + \mu_{pr})[\{I : R\}]_c. \tag{3}$$

The first of these is not the final form for the ribosome concentration. It is given in equation (11) below because we can tie the ribosome concentration directly to the cell density.

3. Population dynamics

The cell population is driven by a logistic growth equation:

$$\frac{dN}{dt} = gN(t)[1 - N(t)/N_0] - \mu_N N(t), \tag{4}$$

where $N(t)$ is the cell population at time t. Here μ_N is the apoptosis rate for the cell species and is to be determined experimentally. Likewise, the number N_0 is the carrying capacity and g is the growth rate.

We assume that the apoptosis rate related to the growth rate by the equation $\mu_N = (1 - \theta)g$. Then we have

$$\frac{dN}{dt} = kN(t)[1 - N(t)/N_m]. \tag{5}$$

where $k = \theta g$ and $N_m = \theta N_0$. One can think of $1/N_m$ as the maximum volume of the live cells that can be supported by the medium subject to the available nutrients in the medium. This number can be determined experimentally.

Notice that in (2) we did not specify $-\mu_r[R]_c + S_{R,ch}$. One way to do this is to make the assumption that there is a constant κ such that $\kappa[N] = [R]_c$, an assumption that is consistent with the experiments we are modeling. (Inhibition of ribosome growth results in inhibition of protein assembly and hence cell division.) Then from equation (5) we have

$$\frac{d[R]_c}{dt} = k[R]_c[1 - [R]_c/R_m] \tag{6}$$

where we have set $R_m = \kappa N_0$. Comparing this with (2) we find that

$$k[R]_c[1 - [R]_c/R_m] = -\mu_r[R]_c + S_{R,ch}. \tag{7}$$

This ties the synthesis rate of the ribosome to the cell density. If we assume that $\mu_r = 0$, then in the absence of inhibitor,

$$\frac{d[R]_c}{dt} = k[R]_c[1 - [R]_c/R_m] = S_{R,ch}. \tag{8}$$

When the inhibitor is present, this is replaced by

$$\frac{d[R]_c}{dt} = k[R]_c[1 - [R]_c/R_m] + k_{-1}[\{I : R\}]_c - k_1[I]_c[R]_c. \tag{9}$$

If we let $[R]_{Tot}(t)$ denote the total ribosome concentration at time t we have

$$[R]_{Tot}(t) = [R]_c(t) + [\{I : R\}]_c(t)$$

while the differential equation for this quantity is

$$\frac{d[R]_{Tot}(t)}{dt} = S_{R,ch}(t) = k[R]_c(t)[1 - [R]_c(t)/R_m]. \tag{10}$$

Then if $[R]_{Tot}(0) = [R]_c(0)$, the total ribosome is simply

$$[R]_{Tot}(t) = [R]_c(0) + \int_0^t S_{R,ch}(s)ds = [R]_c(0) + \int_0^t k[R]_c(s)[1 - [R]_c(s)/R_m]ds. \tag{11}$$

4. Aptamer-inhibitor dynamics

The aptamer-inhibitor chemistry is similar:

$$\{A:I\} \underset{l_1}{\overset{l_{-1}}{\rightleftharpoons}} A+I \tag{12}$$

with l_1, l_{-1} denoting the on and off rates.

The inhibitor enters and leaves the cytoplasm either from (1) the passive diffusion across the membrane, (2) the interaction of the aptamer with the inhibitor either in the cytoplasm or in the cytoplasmic face: or (3) via its interaction with the ribosome or (4) via decay. In particular, the ribosome inability to directly access the cytoplasmic face does not mean that the small back reaction from the membrane pump cannot contribute to ribosome inactivation.

The aptamer is sourced in the chromosome but appears instantaneously in the cytoplasm. Moreover, the aptamer in the cytoplasm can diffuse to and from the cytoplasmic face, (cf) across the juxta-membrane, (j). Thus,

$$\frac{d[A]_c}{dt} = -\frac{V_{A,r}\left([A]_c - [A]_{cf}\right)}{\delta_r} + (l_{-1}[\{I:A\}]_c - l_1[A]_c[I]_c) + S_{A,ch}(t) - \nu_A[A]_c, \tag{13}$$

$$\frac{d[A]_{cf}}{dt} = -\frac{V_{A,r}\left([A]_{cf} - [A]_c\right)}{\delta_r} + (l_{-1}[\{I:A\}]_{cf} - l_1[A]_{cf}[I]_{cf}) - \nu_A[A]_{cf}, \tag{14}$$

where $V_{A,r} = CM_A^{-1/3}/\delta_r = D_A/\delta_r$ and where δ_r is the mean thickness of the inner cell membrane region. (Here M_A is the molecular weight of the aptamer. We have used the Stokes-Einstein equation to relate the diffusion coefficient D_A to the inner cell membrane region permeability rate $V_{A,r}$.)

Likewise the products follow similar dynamics:

$$\frac{d[\{I:A\}]_c}{dt} = -\frac{V_{A,r}\left([\{I:A\}]_c - [\{I:A\}]_{cf}\right)}{\delta_r} + l_1[A]_c\,[I]_c - (l_{-1}+\nu_{P_A})[\{I:A\}]_c, \tag{15}$$

$$\frac{d[\{I:A\}]_{cf}}{dt} = -\frac{V_{A,r}\left([\{I:A\}]_{cf} - [\{I:A\}]_c\right)}{\delta_r} + l_1[A]_{cf}[I]_{cf} - (l_{-1}+\nu_{P_A})[\{I:A\}]_{cf}, \tag{16}$$

where we assume that $D_{\{I:A\}_{cf}} \approx D_A$.

Suppose $\nu_A = \nu_{P_A}$. If we add equations (13), (14), (15), (16) we see that

$$\frac{d[A]_{Tot}(t)}{dt} = S_{A,ch}(t) - \nu_A[A]_{Tot} \tag{17}$$

where

$$[A]_{Tot} = [A]_c + [A]_{cf} + [\{I:A\}]_c + [\{I:A\}]_{cf}.$$

Suppose that $([A]_{Tot}(0) = [A]_c(0))$ then

$$[A]_{Tot}(t) = [A]_c(0)e^{-\nu_A t} + \int_0^t e^{-\nu_A(t-s)} S_{A,ch}(s)ds. \tag{18}$$

5. Connection between the ribosome source and the aptamer source

If $[A]_{Tot}(t) = \lambda[R]_{Tot}(t)$ for all t and some constant λ, using (10) and (17), it follows that $\frac{d[A]_{Tot}(t)}{dt} = \lambda\frac{d[R]_{Tot}(t)}{dt}$ or

$$S_{A,ch}(t) - \nu_A[A]_{Tot}(t) = \lambda S_{R,ch}(t). \tag{19}$$

That is, the net rate of aptamer transcription is proportional to the rate of ribosome transcription.

Conversely, if $\lambda[R]_c(0) = \lambda[R]_{Tot}(0) = [A]_{Tot}(0) = [A]_c(0)$ and equation (19) holds for all $t > 0$, then $[A]_{Tot}(t) = \lambda[R]_{Tot}(t)$ for all $t > 0$.

Equation (19) implies that $S_{A,ch}(t) - \nu_A[A]_{Tot}(t) \geq \lambda S_{R,ch}(t) \geq 0$.

6. A model for the pump

We use a version of a compartment model discussed in [1, 3] to model the pump.

Imagine the cell membrane of thickness δ_m to be situated so that the $x-$axis is normal to the membrane. The interior of the membrane is then the set of points $\{(x,y,z)|0 < x < \delta_m\}$. Suppose T is the transfer protein in the cell membrane and that $[I]_{cf}(t,x,y,z)$ is the concentration of inhibitor in the inner cytoplasmic face ($x = 0$) while $[I]_e(t,x,y,z)$ is the concentration of inhibitor exterior to the cell ($x \geq \delta_m$). Let $[I]_m(t,x,y,z),\ [J]_m(t,x,y,z)$ be the concentrations of the inhibitor in the membrane near $x = 0$ and $x = \delta_m$ respectively. Then the chemical reactions within the membrane are:

$$I_m + T \underset{\kappa_{-m}}{\overset{\kappa_m}{\rightleftarrows}} \{I_m : T\} \underset{\kappa_{-3}}{\overset{\kappa_3}{\rightleftarrows}} \{J_m : T\} \underset{\kappa_{-2}}{\overset{\kappa_2}{\rightleftarrows}} J_m + T. \tag{20}$$

The κ's with positive indices correspond to inhibitor movement out of the cell while the κ's with negative indices correspond to inhibitor movement into the cell.

The pump is viewed as an efflux pump, i.e., one that favors expulsion of the inhibitor from the cytoplasm. In mathematical terms, this view can be realized by the assumption that

$$\min\{\kappa_m, \kappa_3, \kappa_2\} >> \max\{\kappa_{-m}, \kappa_{-3}, \kappa_{-2}\}. \tag{21}$$

Under such circumstances, one can show that the total concentration of inhibitor bound to the pump is fairly small, See Remarkr:boundinhibitor below.

If we regard the concentrations of the intermediates $\{I_m : T\},\ \{J_m : T\}$ as being nearly constant,[1] we obtain from mass action that

$$0 \approx \frac{d[\{I_m : T\}]}{dt} = \kappa_m[I]_m[T] + \kappa_{-3}[\{J_m : T\}] - (\kappa_{-m} + \kappa_3)[\{I_m : T\}]$$

with a similar expression for $d[\{J_m : T\}]/dt$. We obtain the matrix equation

$$[T]\begin{bmatrix}\kappa_m[I]_m \\ \kappa_{-2}[J]_m\end{bmatrix} = \begin{bmatrix}\kappa_{-m} + \kappa_3 & -\kappa_{-3} \\ -\kappa_3 & \kappa_2 + \kappa_{-3}\end{bmatrix}\begin{bmatrix}[\{I_m : T\}] \\ [\{J_m : T\}]\end{bmatrix}$$

or, upon solving the above system of linear equations (written in matrix form), that

$$\begin{bmatrix}[\{I_m : T\}] \\ [\{J_m : T\}]\end{bmatrix} = \frac{[T]}{\mathfrak{d}_\mathfrak{e}}\begin{bmatrix}\kappa_2 + \kappa_{-3} & \kappa_{-3} \\ \kappa_3 & \kappa_{-m} + \kappa_3\end{bmatrix}\begin{bmatrix}\kappa_m[I]_m \\ \kappa_{-2}[J]_m\end{bmatrix} \tag{22}$$

where $\mathfrak{d}_\mathfrak{e} = (\kappa_{-m} + \kappa_3)(\kappa_2 + \kappa_{-3}) - \kappa_{-3}\kappa_3 > 0$. Suppose the total concentration of transfer protein is fixed, T_0 say. Then in the cell membrane, $[T] + [\{I_m : T\}] + [\{J_m : T\}] = T_0$ and consequently: $[T] = T_0/(1 + \mathfrak{K}_2'[I]_m + \mathfrak{K}_{-1}'[J]_m)$ where $\mathfrak{K}_2' = (\kappa_2 + \kappa_3 + \kappa_{-3})\kappa_m$ and $\mathfrak{K}_{-1}' = (\kappa_{-m} + \kappa_3 + \kappa_{-3})\kappa_{-2}$. Thus, from mass-action we obtain:

$$\begin{aligned}\frac{d[I]_m}{dt} &= -\kappa_m[I]_m[T] + \kappa_{-m}[\{I_m : T\}] = -(\kappa_m\kappa_2\kappa_3[I]_m - \kappa_{-m}\kappa_{-2}\kappa_{-3}[J]_m)[T]/\mathfrak{d}_\mathfrak{e} \\ &= \frac{-(\kappa_m\kappa_2\kappa_3[I]_m - \kappa_{-m}\kappa_{-2}\kappa_{-3}[J]_m)T_0}{\mathfrak{d}_\mathfrak{e} + \mathfrak{K}_2'[I]_m + \mathfrak{K}_{-1}'[J]_m}.\end{aligned}$$

To relate the concentrations inside the membrane to those in the cytoplasmic face and in the cell exterior we write $[I]_m = K_I[I]_{cf}$ and $[J]_m = K_{I_e}[I]_e$ where the $K's$ are the partition coefficients.

[1] This is the Michealis-Menten hypothesis. See [2].

Then with the understanding that $\mathfrak{K}_2 = K_I\mathfrak{K}_2'$ and $\mathfrak{K}_{-1} = K_{I_e}\mathfrak{K}_{-1}'$, at $x = 0$ we have

$$\frac{d[I]_{cf}}{dt} = \frac{-(\kappa_m\kappa_2\kappa_3[I]_{cf} - \kappa_{-m}\kappa_{-2}\kappa_{-3}(K_{I_e}/K_I)[I]_e)T_0}{\mathfrak{d}_{\mathfrak{c}} + \mathfrak{K}_2[I]_{cf} + \mathfrak{K}_{-1}[I]_e}.$$

Finally, to take into account passive diffusion across the membrane, we add the term $-V_{I,m}([I]_{cf} - [I]_e)/\delta_m$ to the right hand side of this equation. Here $V_{I,m}$ is the membrane permeability constant. (Here the membrane and inner cell membrane region permeabilities are given by $V_{I,m} = CM_I^{-1/3}/\delta_m = D_I/\delta_m$, $V_{I,r} = CM_I^{-1/3}/\delta_r = D_I/\delta_r$.) We also include the decay of the inhibitor, by adding an additional term $-\nu[I]_{cf}$ to the right hand side of the rate equation above. After noting that $\mathfrak{K}_{-1} = K_{I_e}\mathfrak{K}_{-1}'$, at $x = 0$, the rate equation becomes of the inhibitor through the membrane the equation

$$\frac{d[I]_{cf}}{dt} = \frac{-(\kappa_m\kappa_2\kappa_3[I]_{cf} - \kappa_{-m}\kappa_{-2}\kappa_{-3}(K_{I_e}/K_I)[I]_e)T_0}{\mathfrak{d}_{\mathfrak{c}} + \mathfrak{K}_2[I]_{cf} + \mathfrak{K}_{-1}[I]_e} - \frac{V_{I,m}([I]_{cf} - [I]_e)}{\delta_m} - \nu_I[I]_{cf}.$$

We must modify this further to include passive diffusion from the cytoplasm to and from the cytoplasmic face, (cf), across the juxta-membrane and to include the inhibitor-aptamer interaction. The rate at which inhibitor in the cytoplasmic face is driven by the pump mechanism, any passive diffusion from the exterior of the cell, by diffusion from the cytoplasm and by its interaction with the aptamer is given by:

$$\begin{aligned}\frac{d[I]_{cf}}{dt} &= \frac{-K_c([I]_{cf} - \epsilon[I]_e)}{\mathfrak{d}_{\mathfrak{c}} + \mathfrak{K}_2[I]_{cf} + \mathfrak{K}_{-1}[I]_e} - \frac{V_{I,m}\left([I]_{cf} - [I]_e\right)}{\delta_m} - \frac{\rho V_{I,r}\left([I]_{cf} - [I]_c\right)}{\delta_r} \\ &\quad - (l_1[A]_{cf}[I]_{cf} - l_{-1}[\{I : A\}]_{cf}) - \nu_I[I]_{cf},\end{aligned} \tag{23}$$

where

$$K_c = T_0\kappa_m\kappa_2\kappa_3 \quad \text{and} \quad \epsilon = \frac{\kappa_{-m}\kappa_{-2}\kappa_{-3}(K_{I_e}/K_I)}{\kappa_m\kappa_2\kappa_3}.$$

The factor ρ is introduced as a permeability adjustment factor in equation (23) to the diffusion of the inhibitor into the cytoplasm compartment. It is introduced because different membranes may have different permeabilities. That is, it is a correction factor for the Stokes-Einstein equation. Since it is known that the inhibitor is highly charged and has a preference for the cytoplasm membrane, very little of it is able to diffuse into the cytoplasm itself. Therefore, we expect that $\rho < 1$.

We also take into account the back flow or the leakage of inhibitor pumped back into the exterior of the cell: The production of inhibitor in the cell membrane just inside the cell takes the form

$$\begin{aligned}\frac{d[J]_m}{dt} &= -\kappa_{-2}[J]_m[T] + \kappa_2[\{J_m : T\}] = (\kappa_m\kappa_2\kappa_3[I]_m - \kappa_{-m}\kappa_{-2}\kappa_{-3}[J]_m)[T]/\mathfrak{d}_{\mathfrak{c}} \\ &= \frac{(\kappa_m\kappa_2\kappa_3[I]_m - \kappa_{-m}\kappa_{-2}\kappa_{-3}[J]_m)T_0}{\mathfrak{d}_{\mathfrak{c}} + \mathfrak{K}_2'[I]_m + \mathfrak{K}_{-1}'[J]_m}.\end{aligned}$$

Using the partition relations and allowing for back diffusion from the outer boundary of the inner cell membrane region to the cell exterior, we have

$$\frac{d[I]_e}{dt} = \frac{(K_cK_I/K_{I_e})([I]_{cf} - \epsilon[I]_e)}{\mathfrak{d}_{\mathfrak{c}} + \mathfrak{K}_2[I]_{cf} + \mathfrak{K}_{-1}[I]_e} + \frac{V_{I,m}\left([I]_{cf} - [I]_e\right)}{\delta_m}. \tag{24}$$

For the cytoplasm, the inhibitor equation takes the form:

$$\begin{aligned}\frac{d[I]_c}{dt} &= \frac{\rho V_{I,r}\left([I]_{cf} - [I]_c\right)}{\delta_r} - (l_1[A]_c[I]_c - l_{-1}[\{I : A\}]_c) - \nu_I[I]_c \\ &\quad - \{k_1[I]_c\,[R]_c - (k_{-1} + \mu_{p_r})[\{I : R\}]_c\}.\end{aligned} \tag{25}$$

In view of equations (23), (24) and (25), our system of ordinary differential equations consists of the nine equations, namely (2), (3), (13), (14), (15), (16), (23), (24) and (25). This is a complete description of the dynamics in the active compartments (c), (cf) and (e). Thanks to the partition equations, we have no further use of the inhibitor concentrations or their target products in the membrane.

7. Summary of initial conditions

The nine ordinary differential equations discussed must of course be supplemented by nine initial conditions. Six of the nine initial conditions vanish, i.e.,

$$[I]_c(0) = [I]_{cf}(0) = [A]_{cf}(0) = [\{I : A\}]_{cf}(0) = [\{I : A\}]_c(0) = [\{I : R\}]_c(0) = 0.$$

The remaining two initial conditions, $[R]_c(0)$ and $\lambda[R]_c(0) = [A]_c(0)$, must be prescribed. The initial exterior concentration of inhibitor is prescribed as $[I]_e(0)$.

8. Conservation laws

When the inhibitor is initially prescribed (bolus) and has a very long half life, one obtains some conservation laws. Such conservation laws provide useful checks when computing solutions of differential equations numerically.

In all cases we have, for the pump protein, at each time,

$$T_0 = [T]_0 = [T](t) + [\{I_m : T\}](t) + [\{J_m : T\}](t).$$

For the inhibitor in the active compartments ((c), (cf) and (e)) we have

$$[I](t) = [I]_c(t) + [\{I : R\}]_c(t) + [\{I : A\}]_c(t) + [I]_{cf}(t) + [\{I : A\}]_{cf}(t) + [I]_e(t). \quad (26)$$

We assume that we can neglect the concentration of the inhibitor in the juxta membrane (j).

The concentrations of the inhibitor and its products in the membrane ($[I]_m, [J]_m, [\{I_m : T\}]$ and $[\{J_m : T\}]$) have been absorbed in the concentrations of $[I]_{cf}$ and $[I]_e$ in the cytoplasm face and in the cell exterior via the use of the partition equations discussed in Section 6.

9. Fraction control and Growth Rates

To define the fraction control, one views the ribosome concentration $[R]_c(t) = [R]_c(t, I_c(t), A_c(t))$ then one defines the fraction control as

$$F_r(t, I_c(t), 0) = \frac{[R]_c(t, I_c(t), 0)}{[R]_c(t, 0, 0)} \text{ and } F_r(t, I_c(t), A_c(t)) = \frac{[R]_c(t, I_c(t), A_c(t))}{[R]_c(t, 0, 0)}. \quad (27)$$

A third fraction control, that does not involve the untreated cells is defined as

$$F_{r,tr}(t, I_c(t), A(t)) = \frac{F_r(t, I_c(t), A_c(t))}{F_r(t, I_c(t), 0)} = \frac{[R]_c(t, I_c(t), A_c(t))}{[R]_c(t, I_c(t), 0)}. \quad (28)$$

This is the form of fraction control we used in our figures below.

Likewise, as long as the derivatives, $d[R]_c(t, 0, 0)/dt, d[R]_c(t, I_c(t), 0)/dt, d[R]_c(t, I_c(t), A_c(t)/dt$ are positive, we can compare these growth rates in a meaningful way. Suppose the initial value of $[R]_c$ are equal, i.e., $[R]_c(0, I_c(0), A_c(0)) = [R]_c(0, 0, 0)$ and $I(0) = 0$. If, for some interval $[0.\tau]$,

$$\frac{d[R]_c(t, I_c(t), 0)}{dt} < \frac{d[R]_c(t, 0, 0)}{dt}, \quad (29)$$

then we say the inhibitor fails to protect cell growth on $[0, \tau]$. If the inequality is reversed on that interval, the inhibitor protects cell growth. We are not interested in this latter case. However, suppose

$$\frac{d[R]_c(t, I_c(t), A_c(t))}{dt} < \frac{d[R]_c(t, I(t), 0)}{dt} < \frac{d[R]_c(t, 0, 0)}{dt} \quad (30)$$

Table 1. Numerical values used in simulations

Equation	Constants	Values	Notes
(2), (3)	k_1	$1.0\ (\mu M h)^{-1}$	on rate for ribosome $Kd_{\{R:I\}}$
(2), (3)	k_{-1}	$10^{-3}\ h^{-1}$	off rate for ribosome $Kd_{\{R:I\}}$
(2)	μ_r	$0\ h^{-1}$	ribosome decay rate
(3)	μ_{pr}	$0\ h^{-1}$	ribosome:inhibitor decay rate
(5,9)	k	$1.39 h^{-1}$	cell growth rate at infinite dilution
(9)	R_m	$1.5\mu M$	ribosome carrying capacity
(10)	$S_{R,ch}(t)$	See Remark 10.1	ribosome source rate $\mu M/h$
(13)	$S_{A,ch}(t)$	See Remark 10.1	aptamer source rate $\mu M/h$
(19)	λ	$5, 10, 15, 20$	See Remark 10.1
(13), (14)	ν_A	$1.39\ h^{-1}$	aptamer decay rate
(15), (16)	ν_{p_A}	$1.39\ h^{-1}$	aptamer:inhibitor decay rate
(13), (14), (15), (16)	l_1	$1.0\ (\mu M h)^{-1}$	on rate for aptamer $Kd_{\{A:I\}}$
(13), (14), (15), (16)	l_{-1}	$10^{-5}h^{-1}, (10^5 h^{-1})$	low (resp. high) off rates $Kd_{\{A:I\}}$
(13), (14), (15), (16)	$V_{A,r}$	$0, 10^{-4}, 10^{-3}, 10^{-2}, 10^{-1}$	aptamer permeability in cm/h
(23), (24)	$V_{I,m}$	$10^{-4}\ cm/h$	inhibitor-membrane permeability
(23), (25)	$V_{I,r}$	$10^{-4}\ cm/h$	inhibitor-juxta-membrane perm.
(23), (25)	ρ	$\rho = 0.01, 0.1, 1, 10$	permeability factor, $\rho V_{I,r}$
(13), (14), (15), (16)	δ_r	$10^{-4}\ cm$	juxta-membrane thickness
(23), (24)	δ_m	$4(10^{-7})\ cm$	membrane thickness
(23)	T_0	$10\ \mu M$	total available transfer protein
(23)	$\kappa_{-m}, \kappa_{-2}, \kappa_{-3}$	$10^{-3}\ h^{-1}$	pump inflow rates
(23)	$\kappa_m, \kappa_2, \kappa_3$	$1.0\ (\mu M h)^{-1}$	pump outflow rates
(23), (24)	K_{I_e}	1.0	partition coefficient (See text.)
(23), (24)	K_I	1.0	partition coefficient (See text.)
(23), (25)	ν_I	$0\ h^{-1}$	Inhibitor decay rate

on that interval. Then we say the inhibitor protects cell growth better without the aptamer than with it or that cells with the aptamer are less protected from the inhibitor than those without it. However, it could happen that

$$\frac{d[R]_c(t,0,0)}{dt} \geq \frac{d[R]_c(t, I_c(t), A_c(t))}{dt} > \frac{d[R]_c(t, I(t), 0)}{dt}. \tag{31}$$

This can happen if the aptamer is very tightly bound to the inhibitor and the latter is not too much in excess of the former. Then we say that cells with the aptamer are better protected from the inhibitor than those without the aptamer.

10. Numerical results

Below we provide the numerical values we used for our simulations. The figure captions contain cross links to the corresponding figures used in the main body of the paper.

Remark 10.1. The constant λ couples the aptamer and the ribosome source rates through the model assumption (Section 5) that at any time the ratio of total aptamer to total ribosome can be fixed at one of the values of λ given in the table above.

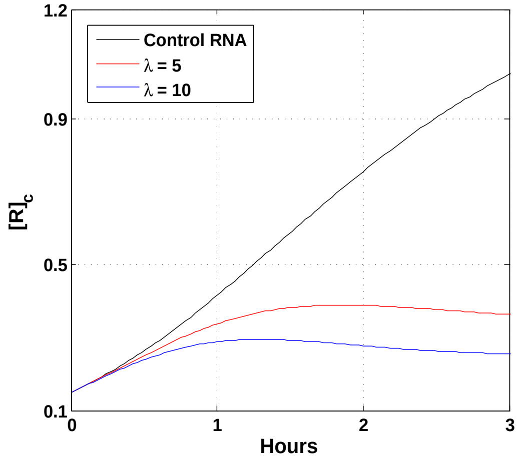

(a) $Kd_{\{A:I\}} = 10^{-4}M$ and $\rho = 0.1$ with $5\mu M$ inhibitor

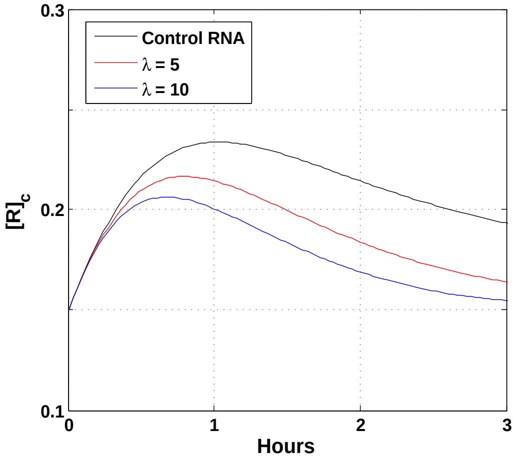

(b) $Kd_{\{A:I\}} = 10^{-4}M$ and $\rho = 1$ with $5\mu M$ inhibitor

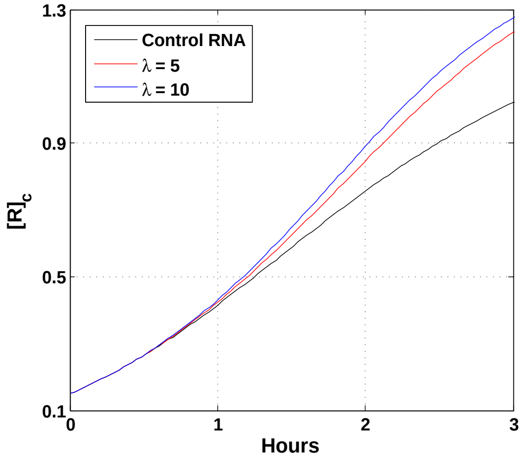

(c) $Kd_{\{A:I\}} = 10^{-9}M$ and $\rho = 0.1$ with $5\mu M$ inhibitor

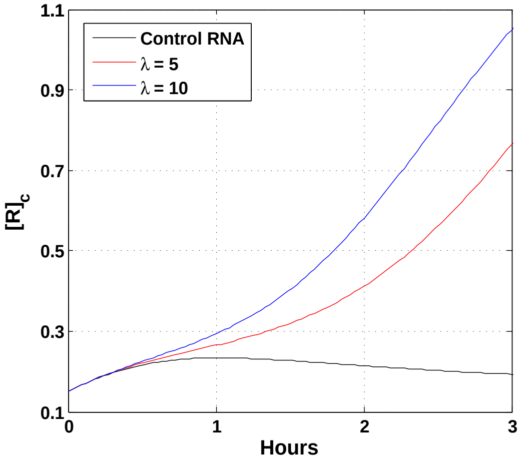

(d) $Kd_{\{A:I\}} = 10^{-9}M$ and $\rho = 1$ with $5\mu M$ inhibitor

FIGURE 1. **The effect of aptamer expression on cell growth.** The initial ribosome concentration in the cytoplasm, $[R]_c(0) = 0.15\mu M$ and $[R]_c$ is proportional to optical density. The net rate of aptamer transcription is proportional to the rate of ribosome transcription, and $[A]_c(0) = \lambda[R]_c(0)$. In comparison with the control, the presence of the aptamer ($\lambda = 5$ or 10) was found to have an effect on cell growth. The values of $Kd_{\{A:I\}} = 10^{-4}M$ (Panels (a,b)) and $Kd_{\{A:I\}} = 10^{-9}M$ (Panels (c,d)) were used in the presence of aptamer. These Kd's correspond to low affinity and high affinity aptamers respectively. In the case of the lower affinity aptamer, the less the inhibitor is able to penetrate the juxta-membrane (smaller ρ) the more protection there is against it. For either value of the permeability adjustment factor, ρ, the aptamer inhibits ribosome growth, more effectively when the inhibitor is less able to penetrate the juxta-membrane on its own. In the case of the high affinity aptamer, the tight binding of the aptamer to the inhibitor protects the ribosome more than in the control, and this is also true for both values of ρ as one might expect. In the presence of aptamer, the permeability constant was fixed as $V_{A,r} = 10^{-1}\ cm/h$. Figure 2E of the paper was taken from Figure 1(a) above.

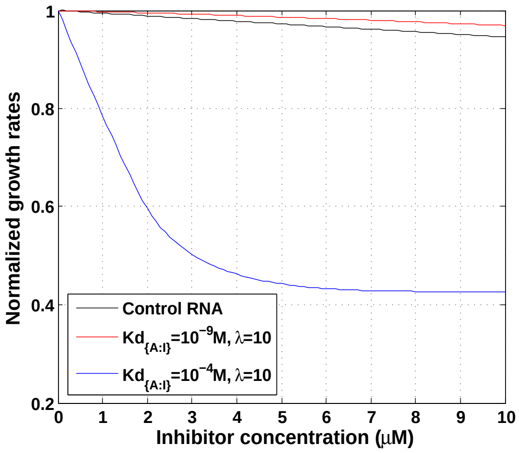

(a) $\rho = 0.01$

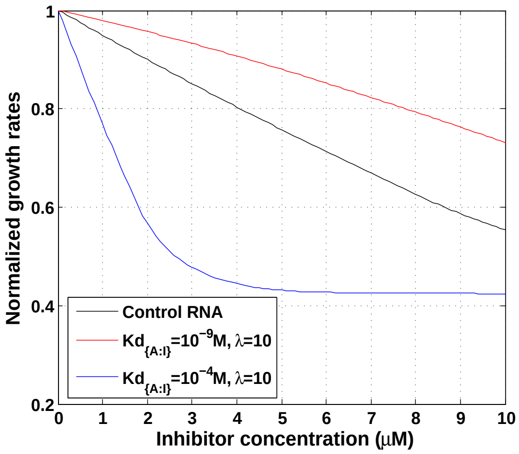

(b) $\rho = 0.1$

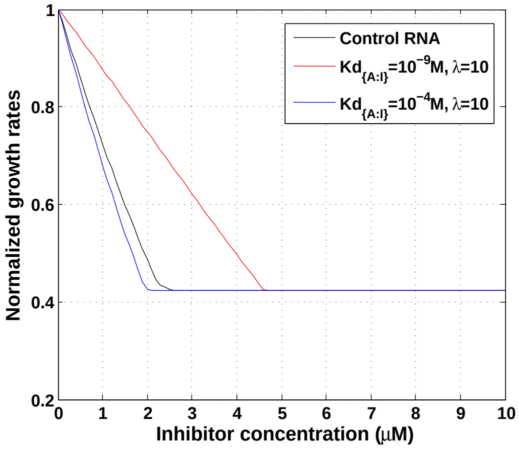

(c) $\rho = 1$

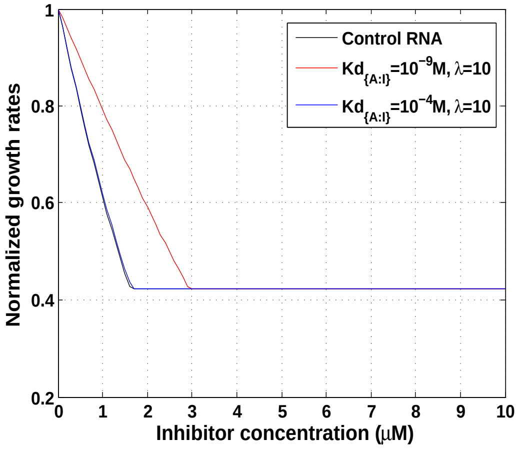

(d) $\rho = 10$

FIGURE 2. **Sensitivity to the permeability adjustment factor, ρ.** The low affinity aptamer is most effective at killing relative to the control when ρ is small and the high affinity aptamer is most affective at protecting relative to the control when ρ is large. In the presence of aptamer, the permeability constant was fixed as $V_{A,r} = 10^{-1}\ cm/h$. Figure 4A of the paper was taken from Figure 2(b) above.

References

1. MAGNARIN, M., MORELLI, M., ROSATI, A., BARTOLI, F., CANDUSSIO, L., GIRALDI, T., AND G. DECORTI, Induction of proteins involved in multidrug resistance (P-glycoprotein, MRP1, MRP2, LRP) and of CYP 3A4 by rifampicin in LLC-PK1 cells, European Journal of Pharmacology, **483(1)**, p.19-28, (2004).
2. MURRAY, J., Mathematical Biology: I. An Introduction, Springer-Verlag New York, Third edition, (2002).
3. TRAN, T. T., MITTAL, A., ALDINGER, T., POLLI, J. W., AYRTON, A., ELLENS, H., AND J. BENTZ, The elementary mass action rate constants for P-gp transport for a confluent monolayer of MDCKII-hMDR1 cells, Biophysical Journal, **88**, p.715-738, (2005).

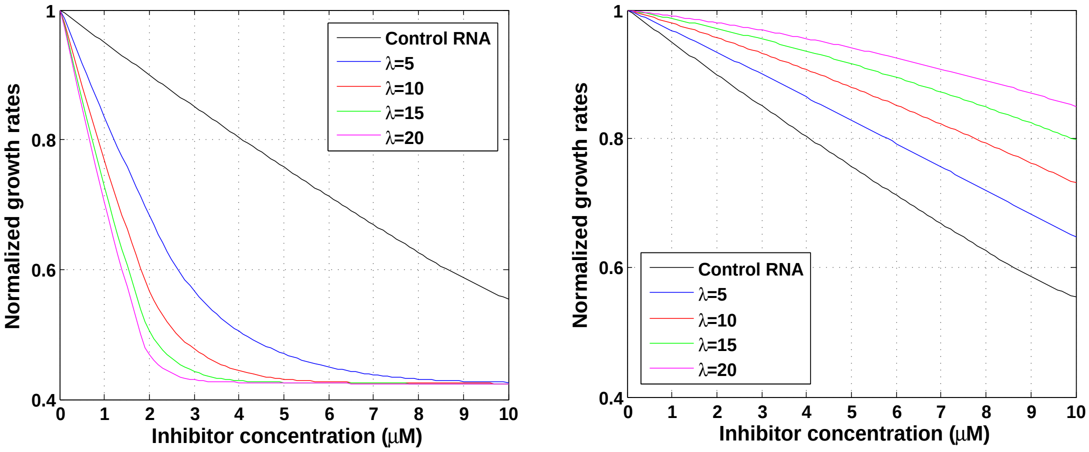

(a) $Kd_{\{A:I\}} = 10^{-4}M$ and $\rho = 0.1$

(b) $Kd_{\{A:I\}} = 10^{-9}M$ and $\rho = 0.1$

FIGURE 3. **Sensitivity to the source rate ratio, λ.** Here we see that increasing the production rate of low affinity aptamer results in more killing relative to the control (less protection) while increasing the production rate of the high affinity aptamer results in greater protection relative to the control. In the presence of aptamer, the permeability constant was fixed as $V_{A,r} = 10^{-1}\ cm/h$. Figure 6B of the paper was taken from Figure 3(a) above.

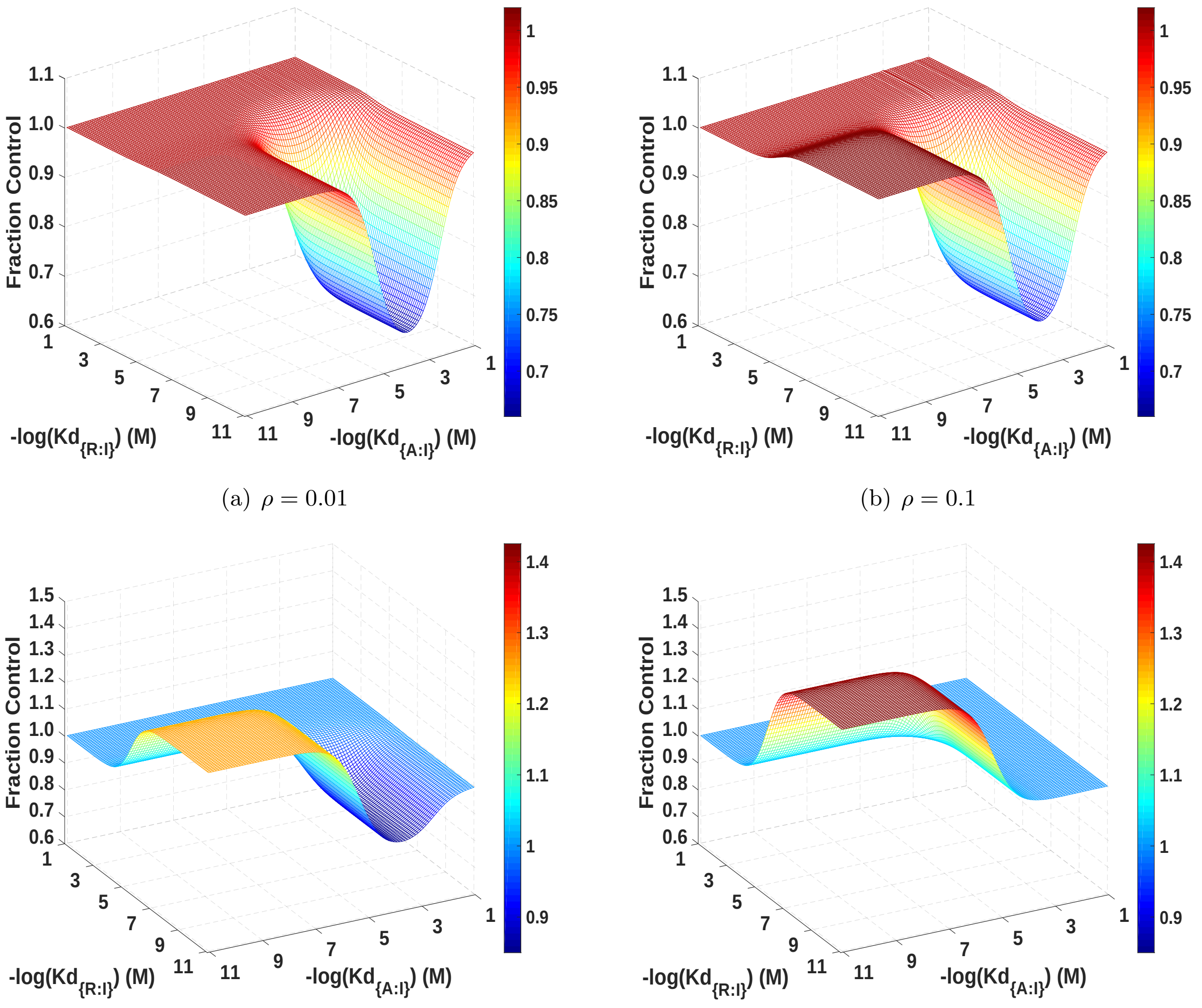

FIGURE 4. **Sensitivity of the model to $Kd_{\{R:I\}}$ and $Kd_{\{A:I\}}$ in the presence of aptamer for various permeability adjustment factors, ρ.** For each panel, the distribution of fraction control (=[free ribosome in the presence of inhibitor and aptamer]/[free ribosome with inhibitor and no aptamer]), after one hour, namely equation (28), was plotted against the negative logarithm of the Kd's for $\{R : I\}$ and $\{A : I\}$ on the $x-$ and $y-$axis, for fixed ρ. The units of Kd's for $\{R : I\}$ and $\{A : I\}$ are molarity, M. In Panels (a) $\sim$ (d), the initial inhibitor concentration in the exterior was $[I]_e(0) = 5\mu M$. The permeability constants were fixed as $V_{I,m} = 10^{-4}\ cm/h$ and $V_{I,r} = 10^{-4}\ cm/h$, as well as $V_{A,r} = 10^{-1}\ cm/h$ in the presence of aptamer ($\lambda = 10$), for all four panels.

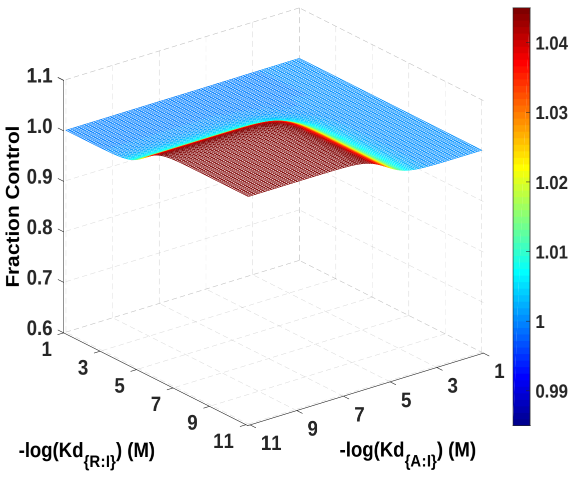

(a) $V_{A,r} = 0\ cm/h$

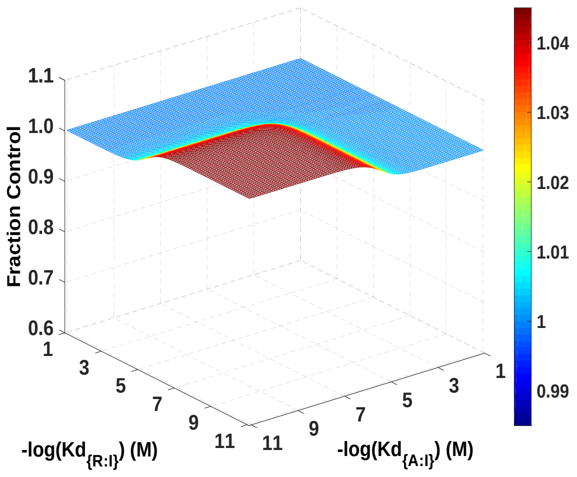

(b) $V_{A,r} = 10^{-4}\ cm/h$

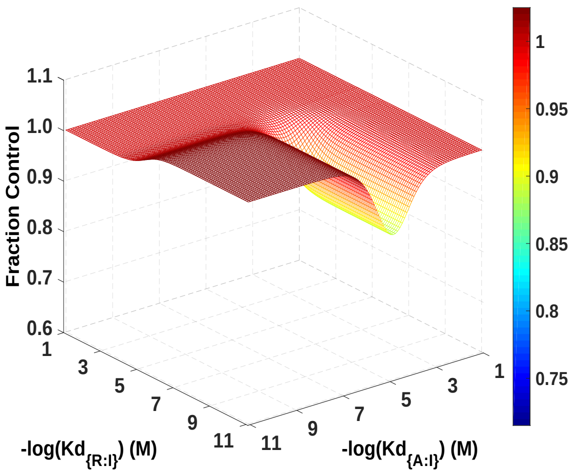

(c) $V_{A,r} = 10^{-3}\ cm/h$

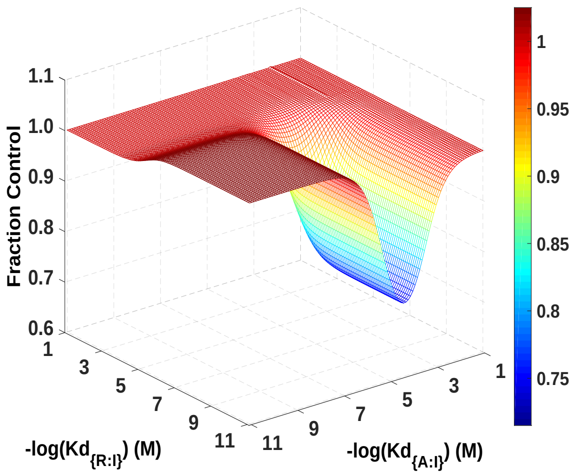

(d) $V_{A,r} = 10^{-2}\ cm/h$

FIGURE 5. **Sensitivity of the model to $Kd_{\{R:I\}}$ and $Kd_{\{A:I\}}$ for various aptamer permeability constants, $V_{A,r}$, when $\rho = 0.1$.** For each panel, the distribution of fraction control (=[free ribosome in the presence of inhibitor and aptamer]/[free ribosome with inhibitor and no aptamer]), after one hour, namely equation (28), was plotted against the negative logarithm of the Kd's for $\{R : I\}$ and $\{A : I\}$ on the $x-$ and $y-$axis, for fixed $V_{A,r}$. In each panel, the initial inhibitor concentration of $[I]_e(0) = 5\mu M$, and the permeability constants of $V_{I,m} = 10^{-4}\ cm/h$ and $V_{I,r} = 10^{-4}\ cm/h$ were also used, as in Figure 4. This experiment demonstrates: If $Kd_{\{R:I\}} < 10^{-5}(M)$ with $10^{-6}(M) < Kd_{\{A:I\}} < 10^{-2}(M)$, the aptamer effect on protection is much less without diffusion than with diffusion, i.e., more killing with diffusion than without it.
Notice that decreasing the aptamer permeability, $V_{A,r}$, has roughly the same effect as increasing the inhibitor permeability factor, ρ. Compare Figures 4(a-d) with figures 5(a-d). Figures 3B, 3C of the paper were taken from Figure 5(a,d).

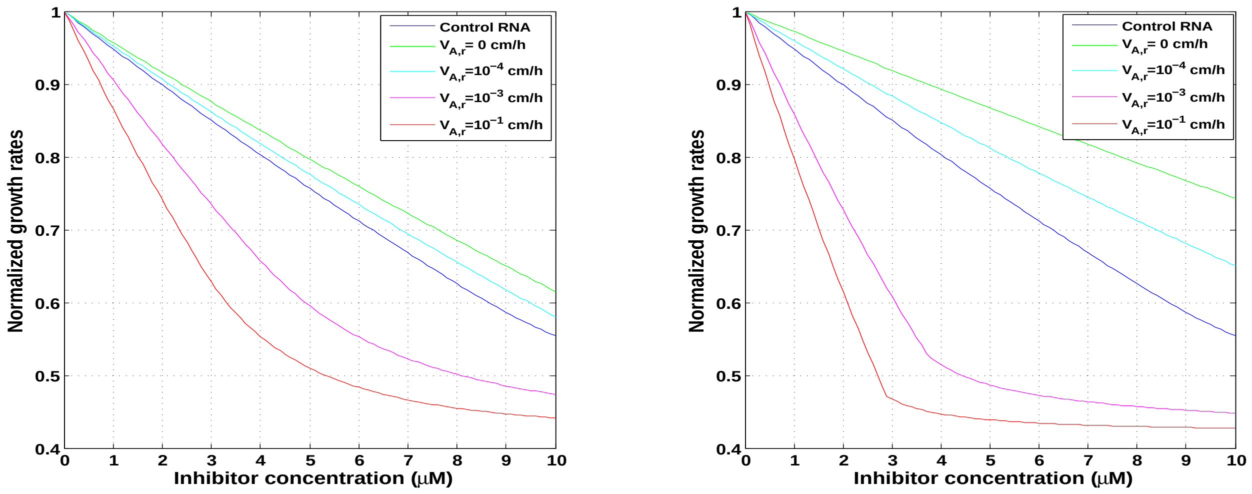

(a) $\lambda = 5$, $\rho = 0.1$ (b) $\lambda = 20$, $\rho = 0.1$

FIGURE 6. **A low affinity aptamer with very low membrane permeability acts as a sink for the inhibitor.** In each panel, the permeability constants of $V_{I,m} = 10^{-4}\ cm/h$ and $V_{I,r} = 10^{-4}\ cm/h$ were used, as in Figure 4. The values of $Kd_{\{R:I\}} = 10^{-9}M$ and $Kd_{\{A:I\}} = 10^{-5}M$ (in the presence of aptamer) were also used in both panels. Figure 6C of the paper was taken from Figure 6(b) above for the cases, $V_{A,r} = 0$ (aptamer with no access), $V_{A,r} = 10^{-1}$ (aptamer with access), and control.

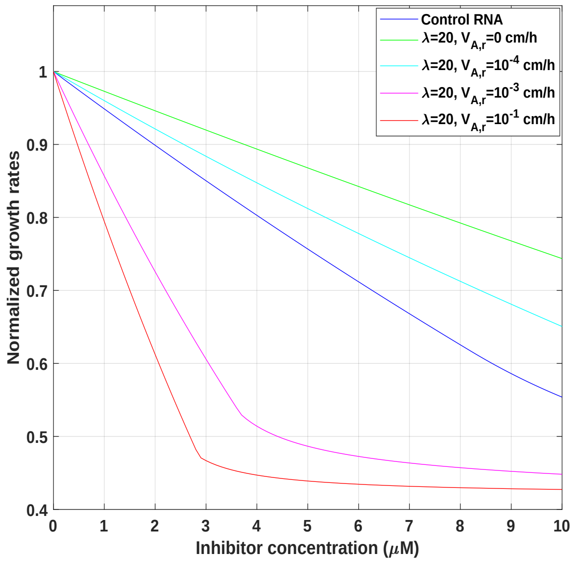

(a) Pump concentration $T_0 = 0.0\ \mu M$

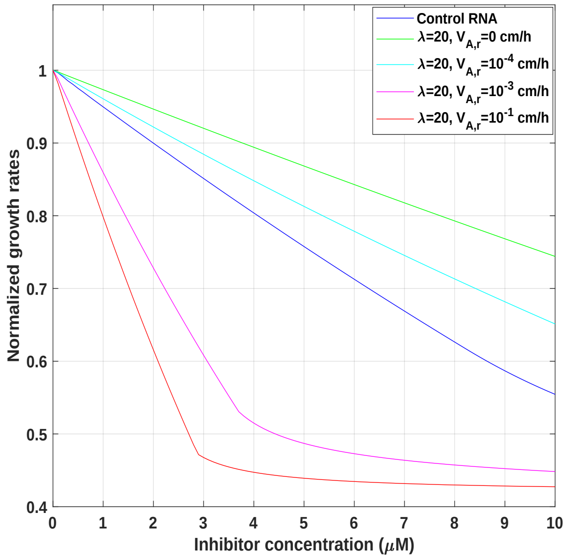

(b) Pump concentration $T_0 = 10.0\ \mu M$

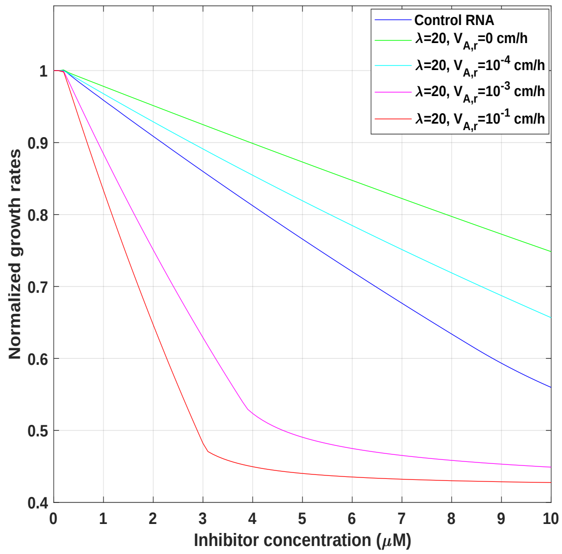

(c) Pump concentration $T_0 = 100.0\ \mu M$

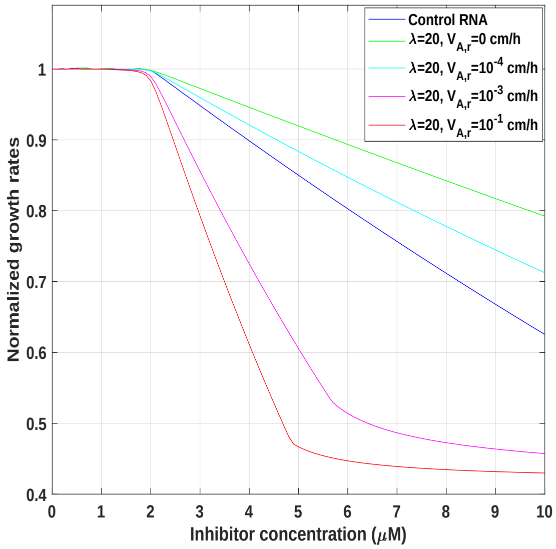

(d) Pump concentration $T_0 = 1000.0\ \mu M$

FIGURE 7. **Test of pump action on low affinity aptamer sink effect in Figure 6(b).** In this set of subfigures, we varied the pump concentration using Figure 6(b) (Figure 7(b)) as a comparison figure. The choice we took for the on and off rates (Table 1) means that the pump is 99.9% efficient in keeping the inhibitor from the ribosome accessible region in the cell cytoplasm. We see that as the pump concentration is increased, we lose the sink effect for very small concentrations of inhibitor. This loss is small in Figure 7(c) but increases to just under a concentration of $2\mu M$ for the inhibitor in Figure 7(c). Once the pump action is at capacity, the sink effects of the aptamer return. Figures 7(c), 7(d) show that the pump protects the ribosome if the former is present in sufficiently large concentrations and the external inhibitor concentration is relatively small.
